# How Big of a Problem is Analytic Error in Secondary Analyses of Survey Data?

**DOI:** 10.1371/journal.pone.0158120

**Published:** 2016-06-29

**Authors:** Brady T. West, Joseph W. Sakshaug, Guy Alain S. Aurelien

**Affiliations:** 1 Survey Research Center, Institute for Social Research, University of Michigan-Ann Arbor, Ann Arbor, Michigan, United States of America; 2 Cathie Marsh Institute for Social Research, University of Manchester, Manchester, England; 3 Institute for Employment Research, Nuremberg, Germany; 4 Joint Program in Survey Methodology, University of Maryland-College Park, College Park, Maryland, United States of America; University of Amsterdam, NETHERLANDS

## Abstract

Secondary analyses of survey data collected from large probability samples of persons or establishments further scientific progress in many fields. The complex design features of these samples improve data collection efficiency, but also require analysts to account for these features when conducting analysis. Unfortunately, many secondary analysts from fields outside of statistics, biostatistics, and survey methodology do not have adequate training in this area, and as a result may apply incorrect statistical methods when analyzing these survey data sets. This in turn could lead to the publication of incorrect inferences based on the survey data that effectively negate the resources dedicated to these surveys. In this article, we build on the results of a preliminary meta-analysis of 100 peer-reviewed journal articles presenting analyses of data from a variety of national health surveys, which suggested that analytic errors may be extremely prevalent in these types of investigations. We first perform a meta-analysis of a stratified random sample of 145 additional research products analyzing survey data from the Scientists and Engineers Statistical Data System (SESTAT), which describes features of the U.S. Science and Engineering workforce, and examine trends in the prevalence of analytic error across the decades used to stratify the sample. We once again find that analytic errors appear to be quite prevalent in these studies. Next, we present several example analyses of real SESTAT data, and demonstrate that a failure to perform these analyses correctly can result in substantially biased estimates with standard errors that do not adequately reflect complex sample design features. Collectively, the results of this investigation suggest that reviewers of this type of research need to pay much closer attention to the analytic methods employed by researchers attempting to publish or present secondary analyses of survey data.

## Introduction

Secondary analyses of survey data sets collected from large probability samples of persons or establishments further scientific progress in many academic fields, including (but not limited to) education, sociology, and public health. The samples underlying these data sets, while enabling inferences about population characteristics or relationships between variables of interest in a finite population of interest, are often “complex” in nature, employing sampling strategies such as stratification of the population and cluster sampling [1–2]. These complex sample design features improve the cost efficiency of survey data collection, but also require secondary analysts to employ approaches that account for the effects of the complex sampling statistically [3].

Unfortunately, many secondary analysts of these data sets do not have formal training in survey statistics, and may apply incorrect analytic methods when analyzing these data sets as a result. The application of standard statistical methods to these data sets can lead to incorrect population inferences, which effectively negates the resources dedicated to the survey data collection. This potential *analytic error* on the part of secondary analysts defines an important part of the widely-researched Total Survey Error (TSE) framework [4–8]. Unfortunately, this important component of TSE has received almost no research attention relative to the other important sources of survey error that define this framework.

In this article, we extend prior knowledge about the magnitude of the analytic error problem by: 1) reviewing representative samples of research products presenting analyses of three different nationally representative survey data sets, to understand the statistical approaches that users of these data employed; 2) identifying evidence of apparent analytic errors in the studies, and quantifying the prevalence of the different types of errors over time across the studies; 3) attempting to isolate sources of the apparent analytic errors based on the dissemination format (formal journal article, book chapter, technical report, conference presentation, etc.); and 4) demonstrating the implications of making analytic errors for inferences based on analyses of survey data. The results of this study suggest that analytic error is a significant problem in these types of research investigations, and these findings have important implications for peer reviewers and the scientific community more generally.

### Alternative Approaches to Survey Data Analysis

There are generally two schools of thought in the survey statistics literature with regard to correct theoretical approaches to the analysis of survey data arising from complex samples [9]. First, the *design-based* analysis approach is characterized by 1) the use of sampling weights for unbiased estimation of parameters describing finite populations (e.g., means, proportions, regression coefficients, etc.), where the weights may be adjusted for survey nonresponse and calibrated to reflect known population features [10], and 2) non-parametric estimation of the variances of weighted estimates using either codes describing complex sampling features (such as sampling stratum codes, or codes describing sampling clusters) or replicate weight variables [11]. The primary historical developments underlying design-based analysis approaches can be found in Neyman [12], Hansen, Hurwitz and Madow [13], Kish [1], Cochran [14], Binder [15] and Korn and Graubard [16]; design-based methods for variance estimation are discussed at length in Wolter [11], Heeringa, West and Berglund [2] and Valliant, Dever and Kreuter [10].

Second, the *model-based* analysis approach ignores the notion of a finite population, and assumes that the survey data arise from an infinite data generation process governed by a probability model, where estimation of the parameters that define that model is the focus of the analysis. Model-based approaches have generally come to rely on various forms of multilevel (or hierarchical linear) models, or Bayesian approaches [17–18]. The complex sampling features essentially become predictors in these models, entering as either fixed effects (for strata that are fixed by design across hypothetical repeated samples) or random effects (for randomly sampled clusters). The analyst also needs to decide whether to use the sampling weights to estimate the parameters of the probability model [19–23], or include the weights as covariates to “control” for the relationships of features used to define the weights with the dependent variable [16]. This decision is not clearly guided by any theoretical results, and has been a source of controversy among statisticians [16, 19, 24, 25, 26].

There are thus alternative “correct” approaches that a secondary user of survey data can take when analyzing complex sample survey data. Recent publications have even attempted to unite these two broader types of approaches into single analytic paradigms [24, 27, 28]. Unfortunately, analysts of survey data from fields outside of statistics and survey methodology generally do not have the benefit of technical training in these alternative approaches. This lack of training can lead to analytic errors in published analyses of survey data when methods appropriate for “standard” simple random samples (or independent and identically distributed data) that are taught as a critical component of many degree programs are applied when analyzing the data. The key point for analysts is that the sample design features are accounted for, regardless of the approach used. A failure to do this can lead to biased estimates and incorrect inferences [16].

### Contributions to the Existing Literature

The study presented in this article makes several unique contributions to the very small amount of existing literature on analytic error. We build on an initial pilot study of analytic error in 100 published, peer-reviewed journal articles, which found that the failure to use one of the correct analytic approaches described above in published secondary analyses of a variety of public health-related complex sample survey data sets is in fact quite common [29–30]. While the current study also focuses on potential analytic errors in secondary analyses of complex sample survey data, it makes several unique contributions relative to this initial pilot study:

1. We consider the possibility of analytic error in additional types of research products aside from peer-reviewed journal articles, including conference / proceedings papers, technical reports, and book chapters;
2. We draw a formal stratified sample of 145 unique research products, treating different decades as sampling strata, enabling us to assess trends in the types of analysis approaches used across different decades;
3. We consider a sample of research products focused on describing the features of the science and engineering workforce in the U.S., as opposed to the public health studies analyzed in the pilot study;
4. We describe what types of analytic approaches secondary analysts are employing when selected variables describing complex sampling features (e.g., replicate weights) are not included in public-use data files and are only available upon request; and
5. We demonstrate the implications of making different types of analytic errors for the quality (bias and variance) of survey estimates, based on several example analyses of real survey data.

We chose to analyze a sample of research products presenting secondary analyses of three complex sample survey data sets from the Scientists and Engineers Statistical Data System (SESTAT, sponsored by the National Center for Science and Engineering Statistics [NCSES]; see http://www.nsf.gov/statistics/sestat/): the Survey of Doctorate Recipients (SDR), the National Survey of College Graduates (NSCG), and the National Survey of Recent College Graduates (NSRCG). These three SESTAT survey data sets are made available to the public online for secondary analysis, and they each arise from samples with complex designs. This requires secondary users of these data sets to employ appropriate estimation methods accounting for the features of the sample designs when analyzing the data. We specifically chose to focus on the SESTAT surveys for three primary reasons:

1. An established body of literature spanning multiple decades has made use of SESTAT data to describe the characteristics of the U.S. workforce, and these data allow users to make timely inferences about important topics regarding the advanced education of the U.S. workforce and trends in its characteristics. In short, we shift the substantive focus of this study to research aimed at describing the scientific capabilities of the U.S. workforce, rather than public health outcomes (as in the pilot study).
2. The NCSES currently employs a fairly unique mechanism to make design information available to public data users for analysis. Final adjusted sampling weights are provided in all public-use SESTAT data sets, but the necessary replicate weights and design codes for variance estimation purposes are presently only available *upon request*. This distinguishes the SESTAT data sets from the other public health survey data sets analyzed in the initial pilot study [29–30], each of which included all of this design information in their public-use data files. SESTAT data users need to read the online documentation very carefully to understand the need to request data files containing the replicate weights and other design information for variance estimation purposes, and this introduces an increased risk of analytic error due to a failure to fully account for complex sampling features. We wanted to assess what analysts of SESTAT data were doing in their studies, given this somewhat unique mechanism for obtaining the public-use data and sample design information.
3. The NCSES is currently making a concerted effort to improve their documentation and also understand the analytic approaches being employed by public users of NCSES survey data (including SESTAT data). In line with these goals, the NCSES recently called for research proposals aiming to improve the analytic methods employed by SESTAT data users (originally National Science Foundation Program Solicitation 12–545, and now 15–521). The present study was part of this evaluation objective.

## Materials and Methods

### Background: SESTAT

Per the official SESTAT web site, “This integrated data system is a unique source of longitudinal information on the education and employment of the college-educated U.S. science and engineering workforce” (see the web site provided above for more information). Table 1 outlines the complex sampling features associated with each of these three survey programs, in addition to recently updated counts of the unique Google Scholar (GS) links associated with each survey (as a proxy measure of the research activity related to each survey).

**Table 1:** Complex sampling features of the three SESTAT surveys, in addition to the number of Google Scholar (GS) links identified when searching for research using each survey (through October 2015).

Name of Survey	Weights?	Strata?	Cluster Sampling?	Longitudinal?	Data access	GS results
Survey of Doctorate Recipients (SDR)	Yes	Yes	No	Yes	Public use / restricted	1,180
National Survey of College Graduates (NSCG)	Yes	Yes	No	Yes	Public use / Census RDC (for strata info)	719
National Survey of Recent College Graduates (NSRCG)*	Yes	Yes	Yes	No	Public use / restricted	294

* The NSRCG was discontinued after 2010.

At present, the NCSES includes final adjusted sampling weights for estimation purposes in all public-use SESTAT data files. The NCSES also makes replicate weights capturing these essential sample design features available to public users of the SESTAT data *upon request* for design-based variance estimation purposes [31]. Interested readers can consult Valliant, Dever and Kreuter [10] or Wolter [11] for more information on design-based variance estimation using replicate weights. Detailed codes describing sampling strata and sampling clusters, which would be especially important for model-based analysis approaches, are also available via restricted-use agreements. Individuals who request the replicate weights or establish these restricted-use agreements are provided with metadata files describing these replicate weight variables and sample design codes [32]. SESTAT data users also have two additional options for taking complex sampling features into account in their analyses:

1. Use a free online analysis tool that provides correct design-based standard errors based on the replicate weights, for straightforward descriptive and tabular analyses; or
2. Use a generalized variance function (GVF) approach to variance estimation [11], incorporating aggregate design effect information provided for estimates computed using SESTAT data [32–33].

Unfortunately, despite the public availability of this information and the opportunity to access the necessary data for appropriate variance estimation by request or via restricted-use agreements, SESTAT data users may ignore the documentation provided or may not be appropriately alerted to the importance of using these variables for variance estimation if they do not search the SESTAT web site carefully. This could adversely affect the inferences that users make based on these three survey data sets. If only the final sampling weights are used in *design-based* estimation, and the complex sampling features representing stratification and cluster sampling are ignored, variance estimates may be biased, ultimately affecting confidence intervals and tests of significance. Users of the SESTAT data employing *model-based* approaches also need to consider what role these weights and sample design codes will play in the probability models that they specify for their variables.

### Sampling of Research Products

We sampled 50 research products presenting analyses of data from each of the three SESTAT surveys using the following methodology. Within Google Scholar (scholar.google.com), a search term was submitted including the name of the survey in quotations, and the word “analysis” (e.g., “National Survey of College Graduates” analysis). The size of the set of search results (*N*) was then considered as the size of the “population” of related products; for example, 655 links or “citations” were identified (October 2015) when submitting the above search term to Google Scholar. Specific year ranges for the products were specified in Google Scholar to ensure appropriate sorting of the identified products by time (implicit stratification), given that time is likely an important factor in the prevalence of analytic errors. More specifically, we hypothesize that knowledge about (and software enabling) appropriate analytic methods for complex sample survey data has become more widely disseminated in recent years, meaning that we expect time and the prevalence of various errors to be negatively correlated. This implicit stratification of the identified research products by time was done to ensure that we had a representative picture of the analytic error problem across different time periods, considering the lifetimes of each of the SESTAT surveys.

This operation therefore resulted in a list of products that was implicitly stratified by year, and the sampling interval (*k* = *N* / 50) was determined in such a way that one of the 50 products will be selected from each interval. Fifty (50) products were then sampled using systematic sampling based on fractional intervals [1], and a stratified random sampling model was used for making inferences based on the resulting sample, with strata defined by collapsing adjacent intervals. The University of Michigan-Ann Arbor provides its researchers with free online access to JSTOR and nearly all major academic journals, so we did not experience any access problems for the journals in which peer-reviewed journal articles appeared.

**Fig 1:**
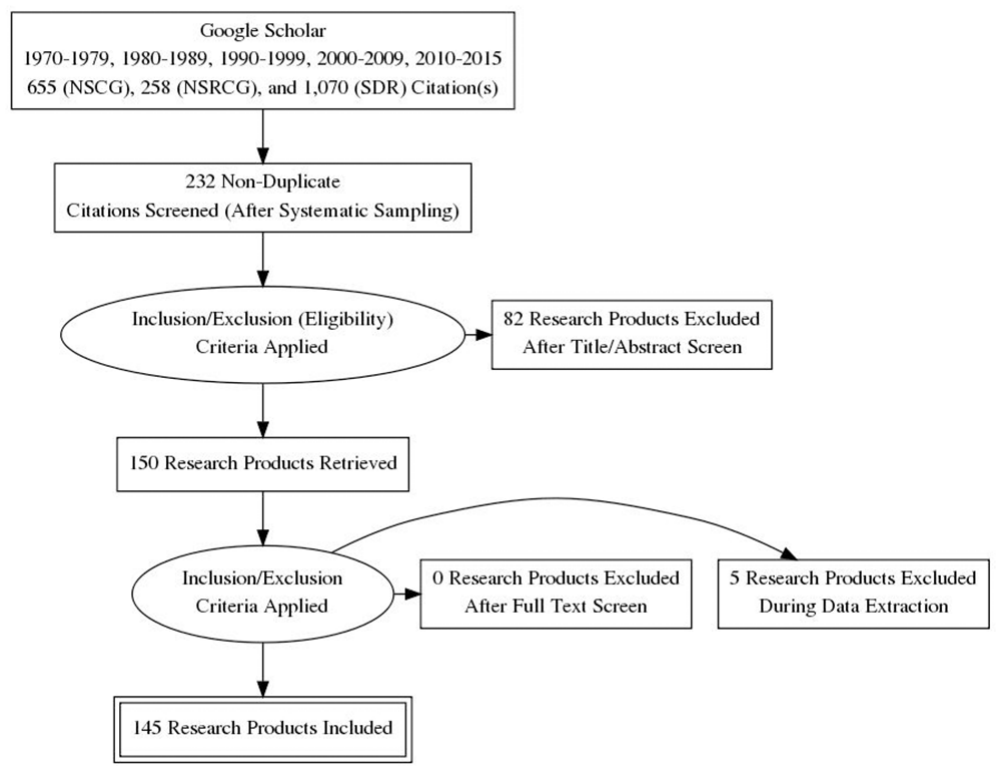
PRISMA Flow Chart Describing Sampling and Screening of Research Products for Meta-Analysis (Generated at http://prisma.thetacollaborative.ca/).

Research products were found to be “eligible” if they actually presented original analyses of the survey data and did not simply refer to other articles presenting analyses of these survey data sets. Products also needed to be readily accessible in electronic format. If 50 “eligible” products were not identified, an additional systematic sample of the required size (e.g., an additional 10 products given 10 ineligible products) was selected. In total, 232 research products were sampled across the three surveys following this procedure, and 82 were excluded based on review of the abstracts (see Fig 1 for the PRISMA flow chart; see also the supporting PRISMA check list in S2 Text). Some products presented analyses of the fully integrated SESTAT database, meaning that data from all three surveys were analyzed simultaneously. These products were only coded once to represent one of the three SESTAT surveys. If some working papers did not provide a date of availability online, we inferred the date based on the most recent cited publication.

A complete reference list for all sampled products can be found in S1 Text. We note that some of the sampled research products were working papers that the authors indicated should not be cited without permission, and we do not cite these papers directly at any point in this study, only reporting results in aggregate.

### Coding Operations

After 50 research products were sampled for each of the three surveys, our research team reviewed and coded each sampled product in a qualitative fashion, recording responses to the following questions:

1. In what year was the product made available for public viewing?
2. Did the analysis account (in some fashion) for the survey weights?
3. Did the analysis account (in some fashion) for the sample design features (e.g., stratification, cluster sampling, replicate weights) in variance estimation?
4. Did the authors appear to use a design-based approach or a model-based approach in the analysis (where the “model-based” approaches include those that ignore sampling features entirely in the model specification)?
5. Did the authors appear to use appropriate statistical software procedures?
6. Did the authors use appropriate methods for subpopulation analysis when design-based methods were employed, per Chapter 4 of Heeringa et al. [2]?
7. How did the authors describe their inferences: with respect to the target population for a given survey (appropriate: e.g., “…an estimated 60% of this population spent four years on their Ph.D.”), or with respect to the sample in hand (inappropriate: e.g., “…60% of the sample spent four years on their Ph.D.”)?
8. Was the sampled product a formal journal article, a book chapter / technical report, or a paper presented at a conference (appearing in conference proceedings)?
9. Relevant features of the journal in which the peer-reviewed article was published (if applicable):
  - Name
  - Web page
  - Peer-review status
  - Impact factor (if available)
  - Does the journal have dedicated statisticians on the Editorial Board, or dedicated statistical reviewers?
  - Does the journal present guidelines (possibly on its web site) for the analysis of survey data?
  - Does the journal have a word limit? If so, what is it?

We systematically recorded answers to each of these questions for each of the 150 sampled research products, and then coded the answers into binary indicators of the various approaches used (e.g., “used design-based approach” or “ignored sample design features in variance estimation”). Two (2) of the 50 sampled SDR products and three (3) of the 50 sampled NSRCG products were found to be general SESTAT review articles upon reading the text in detail, and we were unable to find additional “eligible” SDR or NSRCG products from the same time periods as these five (5) sampled products in a detailed search of the available literature. This resulted in a sample of 145 research products for analysis (see Fig 1 above). Indeed, many of the unique Google Scholar links presented in Table 1 were for research products that *referenced* analyses of data from these three surveys, but did not formally present analyses of data from these surveys (i.e., these research products were ineligible for this study). The appropriate codes for each of the 145 products were reviewed and agreed upon by the entire research team, each of whom reviewed all of the products.

The final data set of coded articles analyzed in this study (available in Excel format; see S1 Data represents a body of evidence with regard to analytic approaches that have been employed by analysts of SESTAT data sets since their initial public availability. We note that in assigning these codes, we are not classifying particular approaches as “correct” or “incorrect,” but rather painting an empirical picture of the types of approaches that tend to be described in research products by analysts of these data. Evidence of consistent failure to account for sample design features in the analyses would suggest potentially high prevalence of analytic errors in these products.

### Statistical Analyses

We employed standard descriptive techniques for estimation based on stratified random samples to compute estimates of the prevalence of each type of analytic approach (both overall and for each survey), in addition to standard errors of the estimates (based on the stratified random sampling model). We also generated descriptive plots indicating the estimated prevalence of each type of analytic approach as a function of the year of publication, for each of the three survey programs. We specifically focused on publication *decades* (e.g., 2000 or earlier, 2001–2010, etc.) when assessing the trends.

We then fit logistic regression models to these data, where individual products are grouped within the individual surveys. The binary indicator for a given type of analytic approach (e.g., using the sampling weights in estimation) was the dependent variable in these models, and the models included fixed effects of publication year (mean-centered within each survey) and possibly other functional forms of publication year (depending on the observed trends). The models also included fixed effects of survey name and interactions between the survey name and year (to determine whether the prevalence of particular approaches is changing over time in a different fashion for the different surveys). The models were fitted using the -logit- command in the Stata software (Version 14+). These trend analyses were purely exploratory; we did not have any *a priori* expectations with regard to the variance in prevalence or trends between the SESTAT surveys. Given the recent proliferation of software for analyzing survey data and quality references on the topic, we did expect to see an overall decrease in the prevalence of analytic errors as a function of publication year; this was suggested by West et al. [29]. We also examined relationships between the type of product (book chapter / technical report, conference presentation / proceedings paper, or formal journal article) and the prevalence of each type of error, separately for each survey and overall.

Next, for the peer-reviewed journal articles, we examined the relationships of journal-level features with indicators of the various analytic approaches, assessing the relationships of all journal features described above with the binary indicators in an exploratory fashion. Fixed effects of these covariates were added to the logistic regression models fitted to the data recorded from the articles, enabling identification of significant journal-level correlates of the analytic approaches used. We also analyzed the co-occurrence of particular analytic approaches (e.g., failing to use weights and failing to use specialized variance estimation methods). To this end, binary indicators of co-occurrence of the various possible approaches were constructed and then modeled using the same approaches described above for the individual indicators.

Finally, we considered the implications of making analytic errors for inferences related to key variables measured in two of the three SESTAT surveys. We did not consider example analyses of the NSRCG data, as this survey was discontinued in 2010 and absorbed into the NSCG. We focused on possible errors made when using a design-based approach, given that this approach is more widely-used by non-statisticians and more readily available in existing software. We first reviewed the research products that we sampled and worked with NSF program officers affiliated with the two surveys to identify key variables that are frequently analyzed by researchers working with these data, in addition to regression models that may be of substantive interest to researchers. Next, we requested the replicate weights for each of the two surveys from NCSES staff, in addition to documentation describing the use of these replicate weights.

For each of the key variables and models from the two surveys listed in Table 2 below, we then considered three alternative approaches to making inferences about descriptive parameters (means, percentages) and analytic parameters (regression coefficients), which included the calculation of estimated standard errors for the estimated parameters and 95% confidence intervals for the parameters:

1. Fully accounting for the complex sampling features, using the weights in estimation and the replicate weights for variance estimation;
2. Using the weights in estimation and Taylor Series Linearization (TSL) for variance estimation (which recognizes variance in the weights), but *ignoring* the replicate weights (which capture complex sampling features such as stratification) when estimating the variances; and
3. Completely ignoring the complex sampling features.

**Table 2:** Key variables and regression models analyzed from two of the three SESTAT surveys to assess the implications of making analytic errors for inferences related to descriptive and regression parameters.

Name of Survey (Year)	Key Variables (See Public-Use Codebooks for Possible Values)	Regression Models of Interest	Final Weight Variable	Replicate Weight Variables
**Survey of Doctorate Recipients (2010)**	Activity Spent Most Hours on in Principal Job (**WAPRI**); Indicator of Salary Greater than $150K (**SALARP** recoded); Race / Ethnicity (**RACETHMP**); Attended Professional Meetings in Past Year (**PROMTGI**); Major Field of Study for Most Recent Degree (**NMRMEMG**); Job Code for Principal Job (**N2OCPRMG**); Labor Force Status (**LFSTAT**); Hours Worked Per Week (**HRSWKP**)	**Logistic Regression Model**: Predict the probability of salary > $150K as a function of Major Degree Field, Race/Ethnicity, and the Interaction between Major Degree Field and Race/Ethnicity; **Ordinal Regression Model**: Predict Hours Worked per Week as a function of Principal Job, Race/Ethnicity, and the Interaction between Principal Job and Race/Ethnicity	**WTSURVY**	**RW001 to RW104** (available upon request from NCSES, with corresponding documentation)
**National Survey of College Graduates (2010)**	Age (**AGE**); Race / Ethnicity (**RACETHM**);U.S. Citizenship Status (**CTZUSIN**);Highest Degree (**DGRDG**); Annual Salary (**SALARY**); Gender (**GENDER**);Labor Force Status (**LFSTAT**); Primary Job in Science and Engineering (Recode of **N2OCPRMG**); Not Working Due to Disability (**NWILL**); Most Recent Degree in Science and Engineering (Recode of **NMRMEMG**)	**Linear Regression Model**: Predict log-transformed current salary with gender, major degree in science and engineering, and their interaction; **Logistic Regression Model**: Predict the probability of having a science and engineering job as a function of gender, race/ethnicity, and their interaction	**WTSURVY**	**COMBINEREPW1 to COMBINEREPW728** (available upon request from NCSES, with corresponding documentation)

When comparing the results from approach 2) to approach 1), we computed the ratio of the estimated variances to assess the effect of ignoring the replicate weights (and therefore the complex sample design features) on the variance estimate. When comparing the results from approach 3) to approach 1), we estimated both the bias in the unweighted estimate (defined as the difference between the unweighted and weighted estimate, treating the weighted estimate as unbiased) and the overall *misspecification effect* [34] on the variance estimate due to completely ignoring the complex sampling features. All analyses were performed using the SURVEYMEANS, SURVEYFREQ, SURVEYREG, and SURVEYLOGISTIC procedures in the SAS software (Version 9.4; SAS Institute, Cary, NC). The S2 Data file in the supporting information contains the SAS code used to download the public-use SESTAT data files, generate the variables for analysis, and perform all of the analyses.

## Results

For each of the three SESTAT surveys individually (and also across all three surveys), Table 3 presents prevalence estimates based on binary indicators of different analytic approaches employed across all years represented in the samples.

**Table 3:** Prevalence of analytic approaches employed, for each of the three SESTAT surveys and overall, across all survey years.

	SDR (n = 48)	NSCG (n = 50)	NSRCG (n = 47)	Overall (n = 145)
Indicator	% (SE)	% (SE)	% (SE)	% (SE)
Accounted for sampling weights in analyses	60.4% (7.3%)	58.0% (7.2%)	44.7% (6.9%)	54.5% (4.2%)
Accounted for complex sampling in variance estimation	2.1% (2.1%)	6.0% (3.4%)	14.9% (5.3%)	7.6% (2.2%)
Used design-based approach (vs. model-based)	50.0% (7.2%)	76.0% (6.1%)	37.0% (7.0%)	55.6% (4.2%)
Used appropriate* subpopulation estimation [2]	4.2% (4.1%)	8.1% (4.6%)	30.8% (13.1%)	10.7% (3.6%)
Described results with respect to the population (vs. the sample)	91.7% (4.0%)	66.0% (6.8%)	65.2% (6.9%)	74.3% (3.7%)

* Restricted to the subpopulation of research products using design-based approaches.

From Table 3, we see initial evidence of some variance across the surveys in the frequency with which investigators employ certain types of approaches. Research products presenting analyses of the SDR and NSCG data are slightly more likely to use the available sampling weights in estimation, but not more likely to use appropriate variance estimation techniques. Design-based approaches were much more common in NSCG products, and SDR products are more likely to describe results with respect to the larger target population. Overall, we found that the sampling weights available in the public-use data files were accounted for in *only about half* of the sampled research products, and appropriate variance estimation and/or subpopulation estimation was *rarely* used (7.6% of publications and 10.7% of publications using design-based approaches, respectively). Nearly 75% of the sampled publications described results with respect to the population rather than the sample, and while a failure to do this is a relatively minor type of error, this is important when describing inferences arising from these types of analyses.

**Fig 2:**
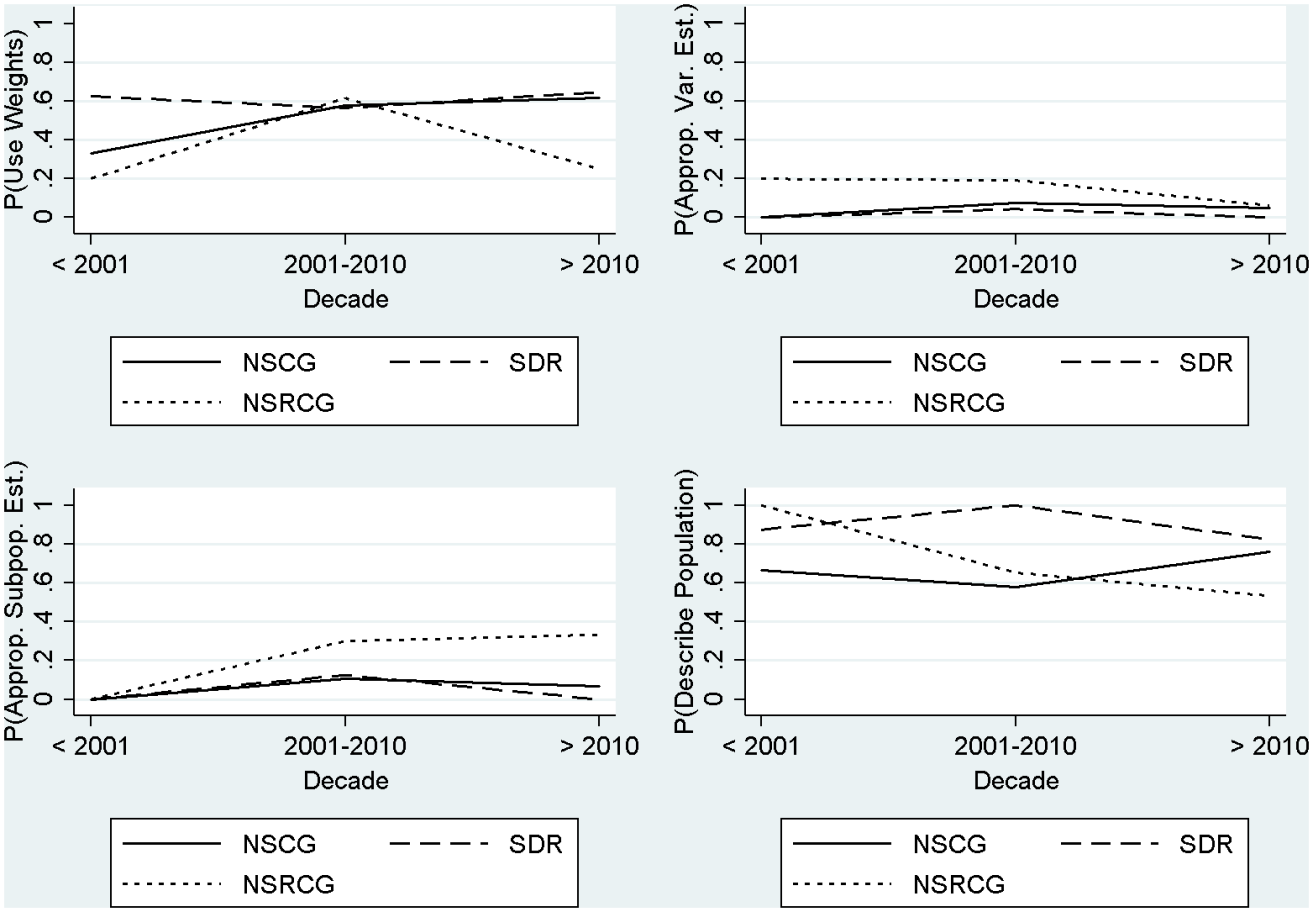
Trends in the prevalence of use of appropriate analytic techniques for secondary analyses of SESTAT data.

Fig 2 presents trends in the prevalence of each of these analytic approaches as a function of the decade in which a sampled research product was first available, for each of the three surveys.

Fig 2 does not present evidence of any significant trends in the prevalence of the different types of analytic approaches over time; that is, the prevalence of using these approaches is fairly stable, centered on the overall estimates for each survey in Table 3. While there is slight evidence of an increase over time in the proportion of research products appropriately using weights in estimation for the NSCG survey (the top-left panel of Fig 2), there do not appear to be consistent trends in the use of appropriate variance estimation (the top-right panel), appropriate subpopulation estimation when design-based approaches are used (the lower-left panel), or descriptions of results with respect to the larger target population (the lower-right panel). In fact, the probability of appropriately describing results with respect to the larger target population (rather than the sample) for the NSRCG is *decreasing* over time.

The plots in Fig 2 suggest that appropriate variance estimation and subpopulation estimation is *rarely performed* across the three surveys, and that the probability of this behavior is not changing over time. Logistic regression models fitted to each indicator confirmed these visual assessments, with no significant decade effects or significant interactions between decade and survey. We did find in these models that when adjusting for decade, the odds of using a design-based approach in the NSCG were more than three times higher compared to the SDR (adjusted odds ratio = 3.1, 95% CI = 1.3–7.4), consistent with what we found in Table 3. We also found that the odds of describing results with respect to the larger target population were nearly six times higher in the SDR when compared to the NSRCG (adjusted odds ratio = 5.8, 95% CI = 1.7–19.1) and more than five times higher when compared to the NSCG (adjusted odds ratio = 5.4, 95% CI = 1.6–17.5), consistent with the results in Fig 2 and Table 3.

Given the findings that users of the NSCG were more likely to employ design-based approaches but *less likely* to describe results with respect to the larger target population, we also examined whether the use of weights in estimation or the use of a design-based approach to the analysis increased the probability of describing results with respect to the target population. Interestingly, when adjusting for decade and survey, the use of weights in estimation and the use of a design-based approach did *not* significantly affect the probability of describing results with respect to the target population (as opposed to the sample). This finding reflects a possible disconnect between the use of appropriate methods and the use of appropriate language to describe the results of these types of analyses.

Table 4 shows the estimated prevalence of each type of error across different types of research products, for each survey. In none of these analyses did we find a significant association between the type of product and the indicator of the approach used, suggesting that the same approaches tend to be used regardless of the type of product. In general, conference papers were the least likely to account for weights in estimation (42.9%), despite being the most likely to use design-based approaches (64.3%). This was an interesting result, and we reviewed what was happening in particular in the case of the NSCG, where there were four sampled conference papers (only one of which used weights in estimation, resulting in the 25% estimate in Table 4). Three of the four sampled conference papers appeared to use a design-based analysis rather than specifying formal models for the variables of interest (i.e., essentially assuming a simple random sample), but two of the three simply failed to mention the use of weights in estimation at any point. Notably, across the three surveys, *published journal articles* were the least likely types of scientific products to perform appropriate variance estimation (4.0%) and perform appropriate subpopulation analyses when design-based approaches were employed (7.1%).

**Table 4:** Prevalence of analytic approaches employed as a function of type of publication, both overall and for each of the three SESTAT surveys, across all survey years.

	SDR (n = 48)			NSCG (n = 50)			NSRCG (n = 47)			Overall (n = 145)		
	Book Chapter / Tech. Report	Conf. Paper	Journal Article	Book Chapter / Tech. Report	Conf. Paper	Journal Article	Book Chapter / Tech. Report	Conf. Paper	Journal Article	Book Chapter / Tech. Report	Conf. Paper	Journal Article
Indicator	%	%	%	%	%	%	%	%	%	%	%	%
Used weights in analyses	78.6%	50.0%	53.6%	72.7%	25.0%	57.1%	45.2%	50.0%	41.7%	58.9%	42.9%	53.3%
Appropriate variance estimation	7.1%	0.0%	0.0%	9.1%	0.0%	5.7%	16.1%	25.0%	8.3%	12.5%	7.1%	4.0%
Design-based approach (vs. model-based)	64.3%	50.0%	42.9%	72.7%	75.0%	77.1%	36.7%	50.0%	33.3%	50.9%	64.3%	57.3%
Appropriate* subpopulation estimation [2]	11.1%	0.0%	0.0%	12.5%	0.0%	7.4%	22.2%	100%	33.3%	15.4%	14.3%	7.1%
Described results with respect to population (vs. sample)	85.7%	100.0%	92.9%	81.8%	50.0%	62.9%	66.7%	25.0%	75.0%	76.4%	64.3%	74.7%

* Restricted to the subpopulation of research products using design-based approaches.

We next assessed bivariate associations between journal-specific factors and each coded indicator variable. First, Table 5 presents some descriptive characteristics of the journals in which peer-reviewed articles were published. We note in Table 5 that the journals in which these articles were published *rarely* provide statistical guidance with regard to analyzing complex sample survey data on their websites or in their submission guidelines, and that less than 50% of the articles (overall) were published in journals with dedicated statisticians on their review boards.

**Table 5:** Prevalence of journal-specific features (peer-reviewed journal articles only).

	SDR (n = 27)	NSCG (n = 34)	NSRCG (n = 12)	Overall
Indicator	% / Mean	% / Mean	% / Mean	% / Mean
Includes dedicated statisticians on editorial board or as reviewers	51.9%	44.1%	25.0%	43.8%
Provides guidelines for survey data analysis	7.4%	0.0%	0.0%	2.7%
Mean Impact Factor*	1.6	1.7	1.8	1.7

* When available.

Second, considering the associations of journal-specific factors with the indicators analyzed above, we found that NSCG articles published in journals with dedicated statistical reviewers were substantially more likely to employ design-based approaches (92.9% vs. 58.8% in journals without dedicated statistical reviewers, p < 0.05), suggesting that statistical reviewers will typically require authors to at least consider design features in their analysis. We also found that all of the published journal articles using appropriate variance estimation techniques and appropriate subpopulation analysis approaches were published in journals with dedicated statistical reviewers. We see these results as motivation for future practice, where forcing authors to think carefully about complex sampling features (regardless of the approach used) may reduce potential analytic errors. This is especially important in light of the finding that journal articles were the *least likely* to use these appropriate variance estimation approaches.

Finally, there were other common issues that emerged when we were reviewing the sampled research products. We often noted references to the presentation of “robust” standard errors (usually in the footnotes of tables), without any additional clarification of how these standard errors were computed. “Robust” standard errors could refer to a number of different types of variance estimators, and simply referring to “robust” standard errors does not clarify whether complex sampling features (such as stratification, which would generally result in more precise estimates) were accounted for in their computation. Furthermore, many of the articles coded as using model-based approaches *did not account for the complex sampling features in any way* in the model specification. When model-based approaches are used, it’s important to make sure that features of the sample designs (e.g., sampling strata in the SDR) are at the very least included in some way in the models, to make the sample design features ignorable in the context of the larger overall estimation objectives. This includes the sampling weights, which were quite often ignored when these “model-based” approaches were employed. Finally, we found that explicit mention of the names of statistical software procedures used to do the analyses was excessively rare (only 5 of the 145 sampled products). This type of information can help to make the analysis approaches used more transparent, and will also help to enable reproducible research.

### Implications of Making Analytic Errors

Before presenting the results of our analyses based on the alternative analytic approaches, we begin with some theoretical expectations to guide our review and interpretation of the results. First, considering the 2010 SDR, the online SESTAT documentation (https://ncsesdata.nsf.gov/doctoratework/2010/sdr_2010_tech_notes.pdf) indicates that a stratified sample design was employed, resulting in unequal probabilities of selection for persons from different sampling strata. Weights were constructed for SDR respondents that reflected the unequal probabilities of selection and also adjustments for differential nonresponse across strata, and replicate weights were constructed that reflected the stratified sample design and also captured uncertainty in the nonresponse adjustments for variance estimation purposes [31–32].

In theory, one would therefore expect that descriptive population estimates based on survey variables with values that vary widely across selected sampling strata subject to oversampling (e.g., those with disabilities and ethnic minorities) would be subject to bias if the SDR respondent weights were ignored in estimation. In terms of variance estimation, the use of the highly variable respondent weights in estimation would be expected to *increase* variance estimates [1], but the stratified sampling would be expected to *decrease* variance estimates for descriptive estimates based on variables with values that vary widely across the strata. This is due to the fact that stratified sampling based on variables that are homogeneous within strata and heterogeneous between strata will increase the precision of survey estimates [1]. Using the weights *only* in estimation (and ignoring the replicate weights for variance estimation purposes) could thus result in increases in variance estimates that would not be offset by the expected gains in precision due to stratified sampling, especially for those variables with values that varied across sampling strata. For SDR variables that are not strongly associated with the sampling strata, the use of weights in estimation and accounting for the replicate weights would generally lead to an increase in variance estimates relative to ignoring the design features entirely. Expected effects of the SDR sample design would therefore depend on the variable being analyzed, but we would expect that using the weights only in estimation may be problematic for the efficiency of estimates based on variables strongly associated with the SDR sampling strata.

Next, considering the 2010 NSCG, a stratified sample design was also employed, only using the American Community Survey (ACS) as a sampling frame [35]. This procedure once again resulted in unequal probabilities of selection for persons from different sampling strata, in part due to oversampling of particular subgroups based on ACS information (e.g., whether or not a person had a science and engineering degree) and the use of probability proportionate to size (PPS) sampling of persons within strata, mainly based on ACS weights [35]. Weights were also constructed for NSCG respondents that reflected the unequal probabilities of selection and adjustments for differential nonresponse across strata, and replicate weights were constructed that reflected the stratified sample design and again captured uncertainty in the nonresponse adjustments for variance estimation purposes [31–32]. We therefore have similar theoretical expectations regarding the effects of ignoring either the weights or the replicate weights on descriptive estimation and variance estimation: estimates based on variables that are related to the NSCG sampling strata (e.g., correlates of having a science and engineering degree) will tend to be biased if the weights are ignored, and the use of replicate weights for variance estimation has the potential to capture gains in sampling efficiency from the stratified sampling and offset some of the increases in the variance of estimates due to the use of the highly variable weights in estimation. We do note that fully accounting for complex sample designs that also feature *cluster sampling* within strata (unlike the SDR and NSCG) when estimating variances, via replicate weights or stratum and cluster codes, would likely *increase* standard errors further relative to the use of weights only, due to the inefficiencies introduced by cluster sampling [2, 11]. This would not be our general expectation here, given the sample designs used for the SDR and the NSCG.

Finally, considering theoretical expectations with regard to the estimation of regression models, the use of the SDR and NSCG weights in estimation will generally lead to unbiased population estimates of regression coefficients. However, the use of weights in estimation could also lead to *inefficient* estimates of regression coefficients (i.e., estimates with standard errors that are excessively large) if a model has been well-specified and the weights do not provide any information about the estimated coefficients [16, 36]. Expected gains in the efficiency of estimates due to stratified sampling would likely not be as large in the case of estimated regression coefficients as in the case of descriptive parameters like means and proportions, given that complex samples are typically designed with descriptive parameters in mind [1]. We remind readers that this is an active area of research, where several methods have been developed to examine whether survey weights should be used when estimating regression coefficients [36]. We examine changes in estimates due to the use of weights in estimation in this section, and whether the stratified sampling does tend to partially offset losses in efficiency due to the use of weights when estimating the regression coefficients of interest.

We now consider some observations related to the estimation of descriptive parameters from the two surveys. For each of the key variables identified in the two surveys (Table 2), Table 6 presents estimates of percentages or means, estimated standard errors of the estimates, and confidence intervals for the descriptive parameters, using the three alternative analytic approaches (the latter two of which involve some form of analytic error). We also include the aforementioned measures of bias in the unweighted estimates, along with the ratios of variance estimates that enable comparisons of estimated variances when fully accounting for the complex sample design versus accounting for the weights only, and when fully accounting for the complex sample design versus ignoring it entirely (the *misspecification effect*, or MEFF).

**Table 6:** Descriptive estimates and inferences related to key variables from the two SESTAT surveys when following alternative analytic approaches.

**SDR 2010: Key Variables (Possible Values)**	**Fully Accounting for Complex Design**			**Accounting for Final Weights Only**				**Ignoring all Complex Sampling Features**				
	**Estimate**	**R-SE**	**95% CI**	**Estimate**	**TSL-SE**	**95% CI**	**Ratio of Variances: R-SE^2^ / TSL-SE^2^**	**Estimate**	**SRS-SE**	**95% CI**	**Bias of Unweighted Estimate**	**MEFF**
**Activity Spent Most Hours on in Principal Job**												
Account. / Fin.	1.06%	0.07%	0.93, 1.19	1.06%	0.07%	0.93, 1.19	1.00	1.05%	0.06%	0.93, 1.17	-0.01	1.36
Basic Research	12.51%	0.21%	12.08, 12.93	12.51%	0.21%	12.10, 12.92	1.00	12.94%	0.20%	12.54, 13.33	0.43	1.10
Appl. Research	19.13%	0.25%	18.65, 19.62	19.13%	0.25%	18.64, 19.62	1.00	19.56%	0.24%	19.08, 20.03	0.43	1.09
Development of Knowledge	6.85%	0.17%	6.51, 7.20	6.85%	0.17%	6.53, 7.18	1.00	6.44%	0.15%	6.15, 6.74	-0.41	1.28
Design	2.56%	0.10%	2.35, 2.76	2.56%	0.10%	2.35, 2.76	1.00	2.40%	0.09%	2.21, 2.58	-0.16	1.23
Computing	3.94%	0.12%	3.71, 4.17	3.94%	0.13%	3.69, 4.19	0.85	3.71%	0.11%	3.48, 3.93	-0.23	1.19
Employee Relations	0.71%	0.05%	0.61, 0.82	0.71%	0.05%	0.61, 0.82	1.00	0.72%	0.05%	0.62, 0.82	0.01	1.00
Managing	14.18%	0.21%	13.76, 14.60	14.18%	0.22%	13.74, 14.62	0.91	14.06%	0.21%	13.65, 14.47	-0.12	1.00
Production	1.11%	0.07%	0.98, 1.24	1.11%	0.07%	0.98, 1.25	1.00	1.03%	0.06%	0.91, 1.15	-0.08	1.36
Services	12.66%	0.22%	12.22, 13.10	12.66%	0.21%	12.24, 13.08	1.10	12.21%	0.20%	11.82, 12.60	-0.45	1.21
Sales	1.70%	0.08%	1.54, 1.87	1.70%	0.08%	1.54, 1.87	1.00	1.60%	0.08%	1.45, 1.75	-0.10	1.00
Quality Management	0.89%	0.06%	0.78, 1.01	0.89%	0.06%	0.77, 1.01	1.00	0.87%	0.06%	0.76, 0.98	-0.02	1.00
Teaching	19.67%	0.24%	19.19, 20.15	19.67%	0.25%	19.17, 20.17	0.92	20.31%	0.24%	19.83, 20.78	0.64	1.00
Other	3.02%	0.09%	2.84, 3.20	3.02%	0.11%	2.81, 3.23	0.67	3.12%	0.11%	2.91, 3.32	0.10	0.67
**Recoded Salary**												
Under $150K	**82.22%**	**0.26%**	**81.71, 82.73**	**82.22%**	**0.25%**	**81.73, 82.71**	1.08	**83.38%**	**0.23%**	**82.94, 83.82**	**1.16**	1.28
$150K +	**17.78%**	**0.26%**	**17.27, 18.29**	**17.78%**	**0.25%**	**17.29, 18.27**	1.08	**16.62%**	**0.23%**	**16.18, 17.06**	**-1.16**	1.28
**Race/Ethnicity**												
Asian Non-Hisp.	18.31%	0.08%	18.16, 18.47	18.31%	0.24%	17.85, 18.77	0.11	18.29%	0.22%	17.86, 18.71	-0.02	0.13
White Non-Hisp.	**74.04%**	**0.09%**	**73.86, 74.21**	**74.04%**	**0.26%**	**73.54, 74.54**	0.12	**67.22%**	**0.26%**	**66.70, 67.74**	**-6.82**	0.12
Minorities	**7.65%**	**0.05%**	**7.55, 7.75**	**7.65%**	**0.13%**	**7.40, 7.90**	0.15	**14.49%**	**0.20%**	**14.10, 14.88**	**6.84**	0.06
**Attended Professional Mtgs. in PY**												
Yes	**60.32%**	**0.31%**	**59.71, 60.94**	**60.32%**	**0.29%**	**59.75, 60.90**	1.14	**61.41%**	**0.27%**	**60.87, 61.95**	**1.09**	1.32
No	**39.68%**	**0.31%**	**39.06, 40.29**	**39.68%**	**0.29%**	**39.10, 40.25**	1.14	**38.59%**	**0.27%**	**38.05, 39.13**	**-1.09**	1.32
**Major Field of Study, Most Recent Degree**												
Computer / Mathematical	7.33%	0.05%	7.22, 7.43	7.33%	0.16%	7.02, 7.63	0.10	7.46%	0.15%	7.17, 7.75	0.13	0.11
Biological Science	23.84%	0.09%	23.67, 24.01	23.84%	0.25%	23.35, 24.33	0.13	24.06%	0.24%	23.59, 24.53	0.22	0.14
Physical Science	17.58%	0.08%	17.42, 17.74	17.58%	0.23%	17.14, 18.03	0.12	17.16%	0.21%	16.74, 17.57	-0.42	0.15
Social Science	26.84%	0.09%	26.67, 27.02	26.84%	0.26%	26.33, 27.36	0.12	26.99%	0.25%	26.50, 27.49	0.15	0.13
Engineering	**17.34%**	**0.08%**	**17.19, 17.50**	**17.34%**	**0.23%**	**16.89, 17.79**	0.12	**16.45%**	**0.21%**	**16.04, 16.85**	**-0.89**	0.15
S & E Fields	**5.20%**	**0.05%**	**5.10, 5.30**	**5.20%**	**0.13%**	**4.95, 5.45**	0.15	**6.02%**	**0.13%**	**5.76, 6.29**	**0.82**	0.15
Non-S & E Fields	1.69%	0.08%	1.53, 1.84	1.69%	0.08%	1.54, 1.84	1.00	1.68%	0.07%	1.54, 1.83	-0.01	1.31
Other	0.17%	0.02%	0.13, 0.22	0.17%	0.02%	0.12, 0.22	1.00	0.17%	0.02%	0.13, 0.22	0.00	1.00
**Principal Job**												
Computer / Mathematical	9.90%	0.14%	9.62, 10.18	9.90%	0.19%	9.52, 10.28	0.54	9.77%	0.18%	9.42, 10.12	-0.13	0.60
Biological Science	18.01%	0.20%	17.62, 18.41	18.01%	0.24%	17.54, 18.49	0.69	18.32%	0.23%	17.86, 18.78	0.31	0.76
Physical Science	11.48%	0.16%	11.16, 11.80	11.48%	0.20%	11.08, 11.88	0.64	11.43%	0.19%	11.06, 11.81	-0.05	0.71
Social Science	18.30%	0.17%	17.98, 18.63	18.30%	0.25%	17.82, 18.79	0.46	18.59%	0.24%	18.13, 19.05	0.29	0.50
Engineering	12.85%	0.17%	12.52, 13.18	12.85%	0.22%	12.42, 13.28	0.60	12.35%	0.20%	11.96, 12.74	-0.50	0.72
S & E Fields	11.39%	0.18%	11.04, 11.73	11.39%	0.20%	10.99, 11.78	0.81	11.60%	0.19%	11.22, 11.98	0.21	0.90
Non-S & E Fields	18.07%	0.25%	17.57, 18.56	18.07%	0.25%	17.58, 18.55	1.00	17.94%	0.23%	17.49, 18.40	-0.13	1.18
**Labor Force Status**												
Employed	**86.03%**	**0.18%**	**85.67, 86.39**	**86.03%**	**0.21%**	**85.62, 86.44**	0.73	**86.76%**	**0.19%**	**86.39, 87.14**	**0.73**	0.90
Unemployed	2.08%	0.08%	1.92, 2.24	2.08%	0.08%	1.92, 2.25	1.00	2.11%	0.08%	1.95, 2.27	0.03	1.00
Not in Labor Force	**11.89%**	**0.18%**	**11.53, 12.24**	**11.89%**	**0.20%**	**11.50, 12.27**	0.81	**11.13%**	**0.18%**	**10.78, 11.48**	**-0.76**	1.00
**Hours Worked Per Week**												
20 or less	6.55%	0.13%	6.29, 6.81	6.55%	0.16%	6.24, 6.86	0.66	6.36%	0.15%	6.07, 6.65	-0.19	0.75
21–35	7.28%	0.18%	6.92, 7.63	7.28%	0.17%	6.95, 7.60	1.12	7.06%	0.16%	6.76, 7.36	-0.22	1.27
36–40	27.95%	0.29%	27.37, 28.53	27.95%	0.29%	27.38, 28.51	1.00	28.12%	0.27%	27.58, 28.65	0.17	1.15
40+	58.22%	0.31%	57.62, 58.83	58.22%	0.32%	57.60, 58.84	0.94	58.46%	0.30%	57.88, 59.05	0.24	1.07
**NSCG 2010: Key Variables (Possible Values)**	**Fully Accounting for Complex Design**			**Accounting for Final Weights Only**				**Ignoring all Complex Sampling Features**				
	**Estimate**	**R-SE**	**95% CI**	**Estimate**	**TSL-SE**	**95% CI**	**Ratio of Variances: R-SE**^**2**^ **/ TSL-SE**^**2**^	**Estimate**	**SRS-SE**	**95% CI**	**Bias of Unweighted Estimate**	**MEFF**
**Mean Age**	**46.29**	**0.09**	**46.11, 46.47**	**46.29**	**0.12**	**46.06, 46.53**	0.56	**46.69**	**0.05**	**46.59, 46.78**	**0.40**	3.24
**Race / Ethnicity**												
Asian, Non-Hisp.	**8.02%**	**0.12%**	**7.78, 8.25**	**8.02%**	**0.20%**	**7.62, 8.41**	0.36	**16.04%**	**0.13%**	**15.77, 16.30**	**8.02**	0.85
Am. Ind./Al. Nat.	0.31%	0.04%	0.24, 0.39	0.31%	0.05%	0.22, 0.40	0.64	0.41%	0.02%	0.37, 0.46	0.10	4.00
Black, Non-Hisp.	**6.69%**	**0.12%**	**6.46, 6.92**	**6.69%**	**0.24%**	**6.23, 7.16**	0.25	**9.17%**	**0.10%**	**8.97, 9.38**	**2.48**	1.44
Hispanic	**6.97%**	**0.14%**	**6.70, 7.24**	**6.97%**	**0.25%**	**6.49, 7.45**	0.31	**9.76%**	**0.11%**	**9.55, 9.97**	**2.79**	1.62
White, Non-Hisp.	**76.34%**	**0.17%**	**76.00, 76.67**	**76.34%**	**0.38%**	**75.59, 77.08**	0.20	**62.20%**	**0.17%**	**61.86, 62.54**	**-14.14**	1.00
Native Hawaii / Pacific Islander	0.27%	0.04%	0.19, 0.35	0.27%	0.05%	0.18, 0.36	0.64	0.40%	0.02%	0.35, 0.44	0.13	4.00
Multiple Race	**1.41%**	**0.09%**	**1.22, 1.59**	**1.41%**	**0.11%**	**1.19, 1.62**	0.67	**2.02%**	**0.05%**	**1.92, 2.12**	**0.61**	3.24
**U.S. Citizen**												
Yes	**94.51%**	**0.15%**	**94.22, 94.79**	**94.51%**	**0.18%**	**94.15, 94.86**	0.69	**92.27%**	**0.10%**	**92.08, 92.46**	**-2.24**	2.25
No	**5.49%**	**0.15%**	**5.21, 5.78**	**5.49%**	**0.18%**	**5.14, 5.85**	0.69	**7.73%**	**0.10%**	**7.54, 7.92**	**2.24**	2.25
**Highest Degree**												
Bachelor’s	**63.33%**	**0.25%**	**62.83, 63.82**	**63.33%**	**0.41%**	**62.51, 64.14**	0.37	**52.64%**	**0.18%**	**52.29, 52.99**	**-10.69**	1.93
Masters	**27.34%**	**0.24%**	**26.87, 27.81**	**27.34%**	**0.38%**	**26.58, 28.09**	0.40	**34.13%**	**0.17%**	**33.79, 34.46**	**6.79**	1.99
Doctorate	**3.51%**	**0.08%**	**3.36, 3.66**	**3.51%**	**0.09%**	**3.32, 3.69**	0.79	**6.88%**	**0.09%**	**6.70, 7.06**	**3.37**	0.79
Professional	5.83%	0.16%	5.51, 6.15	5.83%	0.17%	5.50, 6.16	0.89	6.35%	0.09%	6.18, 6.52	0.52	3.16
**Mean Salary**	**$71,214**	**$685.66**	**69,868.29, 72,560.52**	**$71,214**	**$656.19**	**69,928.28, 72,500.53**	1.09	**$79,380**	**$265.96**	**78,858.79, 79,901.36**	**$8,166**	6.65
**Gender**												
Female	**52.02%**	**0.15%**	**51.72, 52.32**	**52.02%**	**0.45%**	**51.14, 52.91**	0.11	**43.85%**	**0.18%**	**43.50, 44.20**	**-8.17**	0.69
Male	**47.98%**	**0.15%**	**47.68, 48.28**	**47.98%**	**0.45%**	**47.09, 48.86**	0.11	**56.15%**	**0.18%**	**55.80, 56.50**	**8.17**	0.69
**Labor Force**												
Employed	**78.78%**	**0.30%**	**78.18, 79.37**	**78.78%**	**0.36%**	**78.07, 79.49**	0.69	**80.69%**	**0.14%**	**80.41, 80.97**	**1.91**	4.59
Unemployed	**4.26%**	**0.18%**	**3.90, 4.62**	4.26%	0.19%	3.89, 4.63	0.90	4.33%	0.07%	4.19, 4.47	0.07	6.61
Not in Labor Force	**16.96%**	**0.28%**	**16.41, 17.52**	**16.96%**	**0.33%**	**16.32, 17.60**	0.72	**14.98%**	**0.13%**	**14.73, 15.23**	**-1.98**	4.64
**Primary Job**												
S & E	**30.38%**	**0.30%**	**29.80, 30.96**	**30.38%**	**0.39%**	**29.62, 31.13**	0.59	**54.94%**	**0.20%**	**54.55, 55.33**	**24.56**	2.25
Non-S & E	**69.62%**	**0.30%**	**69.04, 70.20**	**69.62%**	**0.39%**	**68.87, 70.38**	0.59	**45.06%**	**0.20%**	**44.67, 45.45**	**-24.56**	2.25
**Not Working due to Disability**												
Yes	1.92%	0.11%	1.69, 2.14	1.92%	0.11%	1.70, 2.13	1.00	1.76%	0.05%	1.66, 1.85	-0.16	4.84
No/Skipped	98.08%	0.11%	97.86, 98.31	98.08%	0.11%	97.87, 98.30	1.00	98.24%	0.05%	98.15, 98.34	0.16	4.84
**Major Degree**												
S & E	**40.51%**	**0.28%**	**39.97, 41.06**	**40.51%**	**0.39%**	**39.74, 41.28**	0.52	**73.35%**	**0.16%**	**73.04, 73.66**	**32.84**	3.06
Non-S & E	**59.49%**	**0.28%**	**58.94, 60.03**	**59.49%**	**0.39%**	**58.72, 60.26**	0.52	**26.65%**	**0.16%**	**26.34, 26.96**	**-32.84**	3.06

R-SE, Design-based standard error using replicate weights; TSL-SE, Taylor Series Linearization standard error, recognizing variance in the weights; SRS-SE, Simple Random Sample Standard Error, ignoring final weights and replicate weights; MEFF, Misspecification Effect on variance estimate, ignoring all complex sampling features [34]; **Boldface cell values**, cases where inferences would change depending on whether one accounted for the complex sampling features.

**Fig 3:**
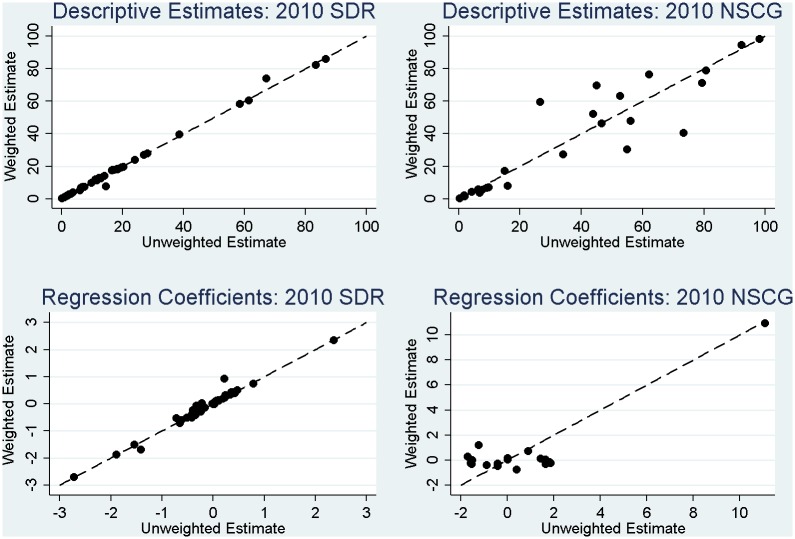
Correspondence between weighted and unweighted estimates of descriptive parameters and regression coefficients in the 2010 SDR and the 2010 NSCG (including a dashed 45-degree line representing perfect correspondence).

The results in Table 6 demonstrate that a failure to account for the complex sample design features in analysis can have severe implications for descriptive estimates and inferences based on those estimates. First, considering the 2010 SDR, a failure to use the final SDR weights in the analysis generally has modest implications for the estimates (see Fig 3), with the most extreme changes noted for race / ethnicity. This was expected in theory, given that these demographic features were used to define sampling strata with different sampling rates. Slight changes in inference are observed for percentages describing distributions of current salary, attending professional meetings in the past year, major fields of Science and Engineering (S & E), and labor force status.

**Fig 4:**
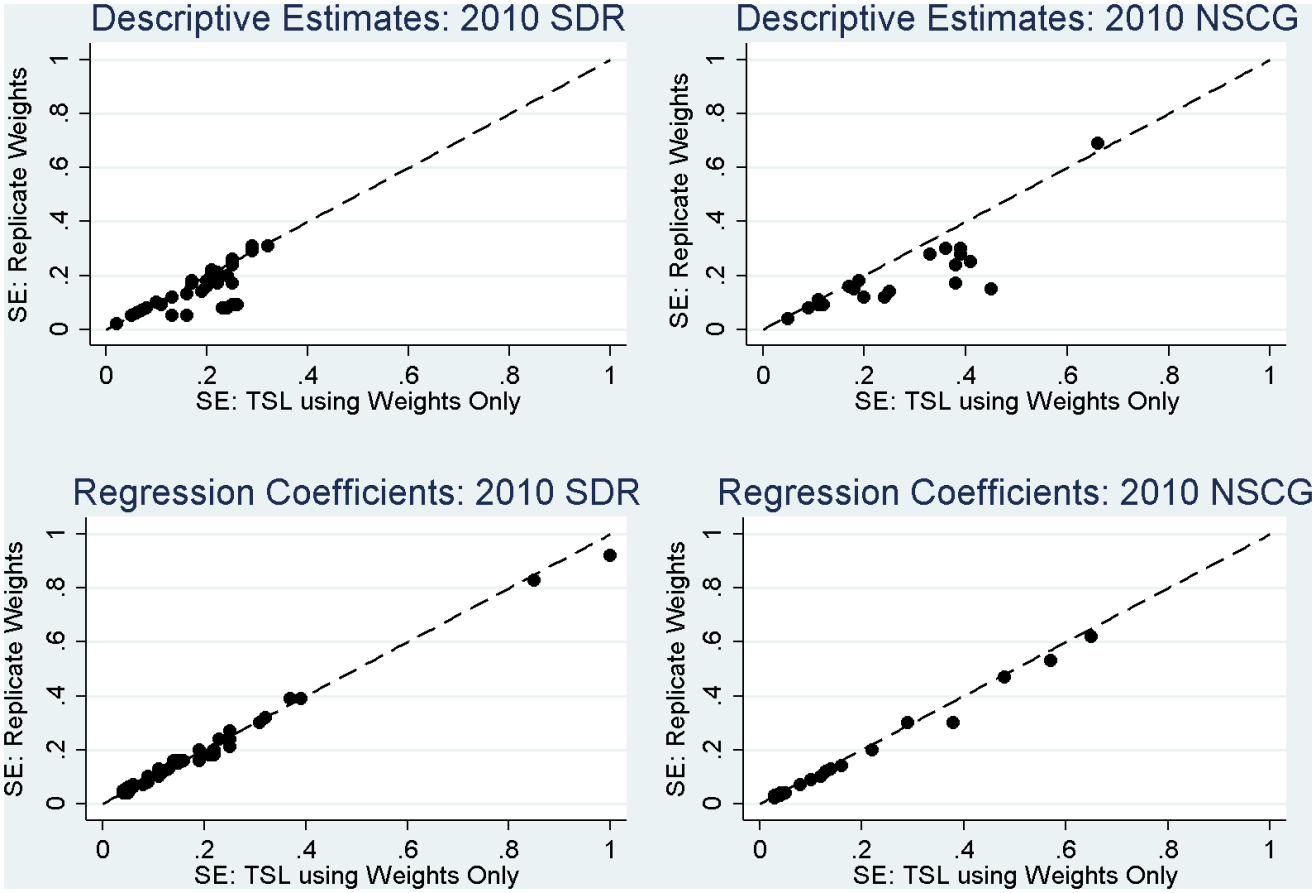
Correspondence between estimated standard errors (SE) for 2010 SDR and 2010 NSCG estimates based on the replicate weights, which fully account for the complex sampling features, and linearized (TSL) standard errors based on the final survey weights only (including a dashed 45-degree line representing perfect correspondence).

More noticeable in the case of the 2010 SDR is consistent evidence of a failure to use the replicate weights in variance estimation leading to variance estimates that tend to be *too large*, as was expected in theory. For a few estimates, use of the replicate weights tends to increase the variance estimates (MEFFs greater than 1), but for most estimates, the replicate weights capture gains in precision of the estimates (MEFFs less than 1) due to the stratified sampling employed in the SDR (Table 1). This pattern is apparent in Fig 4, where the majority of the standard errors for the estimates fall below the 45-degree line. This means that standard errors based on the replicate weights are smaller than standard errors for the same estimates that reflect variance in the survey weights only. These observed gains in efficiency would be *lost* if analysts failed to account for the stratified sampling in variance estimation. Simply using the final weights alone in analysis (in the absence of the replicate weights) does not adequately capture these important gains in efficiency.

**Fig 5:**
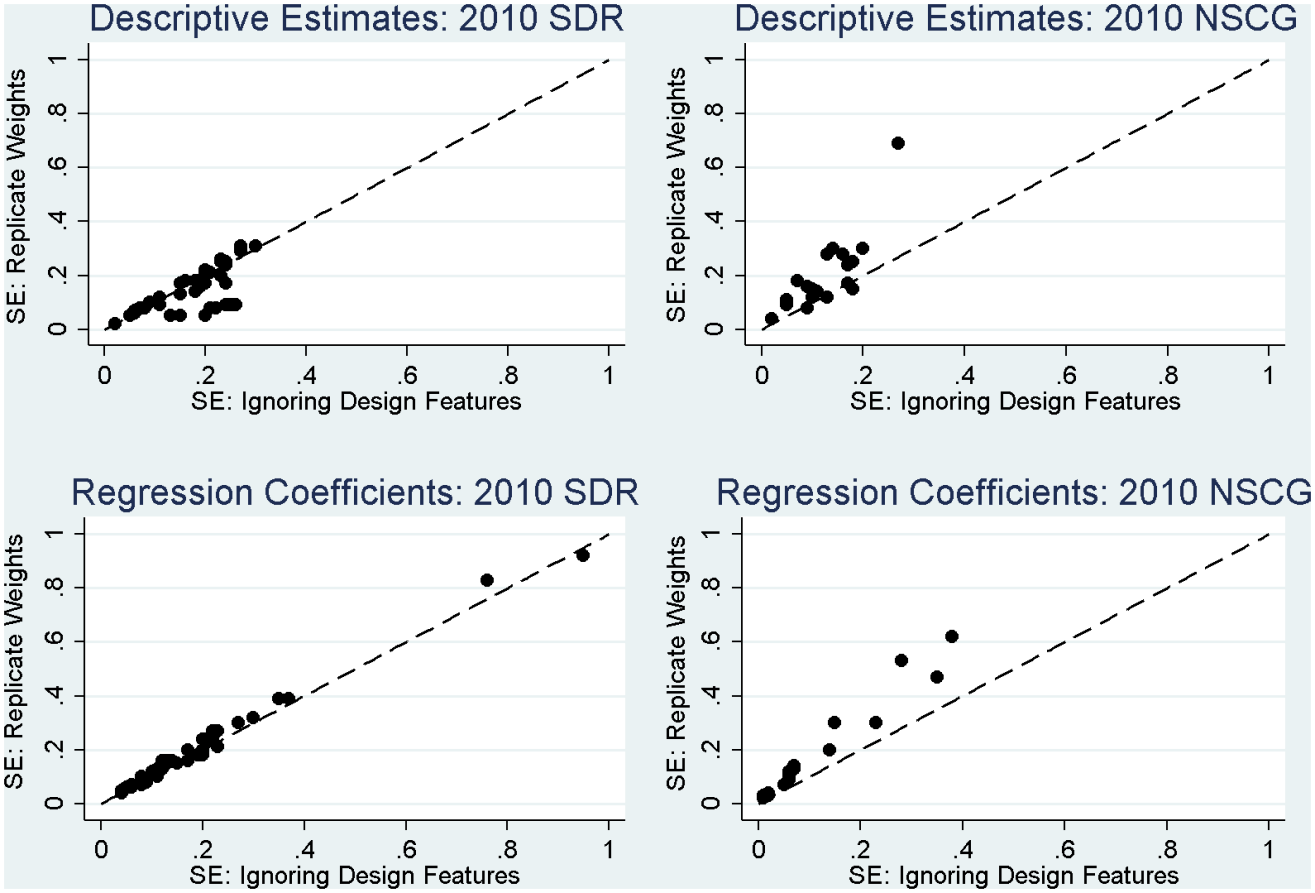
Correspondence between estimated standard errors (SE) for the 2010 SDR and 2010 NSCG estimates based on the replicate weights, which fully account for the complex sampling features, and standard errors based on ignoring the complex sampling features entirely (including a dashed 45-degree line representing perfect correspondence).

Next, considering the estimates for the 2010 NSCG in Table 6, we find that a failure to use the final NSCG weights in estimation has much more severe implications for the resulting estimates relative to the SDR. For the vast majority of the estimates (and especially those related to working in science and engineering fields, as expected), there are substantial changes in the sizes of the estimates when using the weights for estimation (see Fig 3), and inferences would change noticeably regardless of the variance estimation approach employed. These large biases in the unweighted estimates underscore the importance of using the final NSCG weights correctly in estimation; the weights are highly correlated with several of the key measures of interest. Examining the ratios of variances, we note that the misspecification effects tend to be greater than 1, suggesting a general increase in the variance of the estimates that is primarily being driven by the highly variable respondent weights in the NSCG (see Fig 5). However, similar to the SDR analyses, we once again note that a failure to fully account for the stratified sampling (i.e., just using the weights in the analysis) would lead to variance estimates that are *too large*; this pattern is once again evident in Fig 4. While the misspecification effects still tend to be greater than 1 due to the variable respondent weights (Fig 5), a failure to fully account for the stratified sampling would result in variance estimates that were excessively large, and overly conservative inferences.

We now consider the implications of making analytic errors for inferences related to regression model parameters. Table 7 presents estimated regression parameters (along with estimated standard errors and 95% confidence intervals) in the models described in Table 2 for each of the two surveys, following the three different analytic approaches. We also include the aforementioned variance ratios, in addition to design-adjusted multi-parameter Wald tests for the terms included in the models, enabling overall (or omnibus) conclusions about the importance of the terms included in the models (e.g., the overall importance of the major degree field × race/ethnicity interaction in the logistic regression model for the probability of having a salary greater than $150K, based on the 2010 SDR data).

**Table 7:** Estimated regression parameters, standard errors, Wald tests, confidence intervals, and misspecification effects in four regression models fitted to data from the 2010 SDR and NSCG surveys when following the three alternative analytic approaches.

		**Fully Accounting for Complex Design**			**Accounting for Final Weights Only**				**Ignoring all Complex Sampling Features**				
**Survey /Dependent Variable**	**Predictor Variables (Possible Values)**	**Estimated Coefficient**	**R-SE**	**95% CI**	**Estimated Coefficient**	**TSL-SE**	**95% CI**	**Ratio of Variances: R-SE^2^ / TSL-SE^2^**	**Est. Coef.**	**SRS-SE**	**95% CI**	**Bias of Unweighted Estimate**	**MEFF**
**2010 SDR /Binary Indicator of Current Salary > $150K**	Intercept	-1.51	0.07	-1.65, -1.37	-1.51	0.08	-1.67, -1.35	0.77	-1.54	0.08	-1.69, -1.38	-0.03	0.77
	**Major Degree Field**	Wald Test: C-S(7) = 250.55, p < 0.001			Wald Test: C-S(7) = 190.16, p < 0.001				Wald Test: C-S(7) = 199.68, p < 0.001				
	Computer / Mathematical Sciences	0.11	0.10	-0.09, 0.31	0.11	0.11	-0.10, 0.33	0.83	0.07	0.11	-0.14, 0.28	-0.04	0.83
	Biological / Agricultural / Environmental Life Sciences	0.04	0.08	-0.11, 0.18	0.04	0.09	-0.14, 0.21	0.79	0.02	0.09	-0.15, 0.20	-0.02	0.79
	Physical Sciences	0.13	0.08	-0.02, 0.28	0.13	0.09	-0.05, 0.31	0.79	0.11	0.09	-0.07, 0.29	-0.02	0.79
	Social Sciences	-0.26	0.08	-0.41, -0.10	-0.26	0.09	-0.44, -0.08	0.79	-0.26	0.09	-0.44, -0.09	0.00	0.79
	Engineering	0.43	0.08	0.26, 0.59	0.43	0.09	0.24, 0.61	0.79	0.42	0.09	0.24, 0.60	-0.01	0.79
	Non-S & E Fields	0.74	0.15	0.45, 1.02	0.74	0.15	0.44, 1.04	1.00	0.79	0.15	0.50, 1.08	0.05	1.00
	Other Categories	2.34	0.39	1.58, 3.09	2.34	0.37	1.61, 3.07	1.11	2.36	0.37	1.63, 3.08	0.02	1.11
	**Race / Ethnicity**	Wald Test: C-S(2) = 5.58, p = 0.062			Wald Test: C-S(2) = 6.87, p = 0.032				Wald Test: C-S(2) = 6.34, p = 0.042				
	Asian Non-Hisp.	0.19	0.16	-0.13, 0.51	0.19	0.19	-0.19, 0.57	0.71	0.19	0.17	-0.15, 0.53	0.00	0.89
	Minorities	-0.43	0.20	-0.81, -0.04	-0.43	0.19	-0.80, -0.05	1.11	-0.34	0.17	-0.68, -0.01	0.09	1.38
	**Major Degree x Race / Ethnicity**	**Wald Test: C-S(14) = 27.14, p = 0.019**			Wald Test: C-S(14) = 16.60, p = 0.278				Wald Test: C-S(14) = 19.72, p = 0.139				
	Computer / Mathematical Sciences x Asian	-0.71	0.21	-1.11, -0.31	-0.71	0.25	-1.20, -0.22	0.71	-0.65	0.23	-1.09, -0.20	0.06	0.83
	Computer / Mathematical Sciences x Minority	-0.09	0.30	-0.67, 0.49	-0.09	0.31	-0.69, 0.52	0.94	-0.23	0.27	-0.76, 0.29	-0.14	1.23
	Biological / Agricultural / Environmental Life Sciences x Asian	-0.51	0.18	-0.87, -0.15	-0.51	0.22	-0.94, -0.08	0.67	-0.50	0.20	-0.88, -0.12	0.01	0.81
	Biological / Agricultural / Environmental Life Sciences x Minority	-0.07	0.24	-0.54, 0.40	-0.07	0.23	-0.53, 0.39	1.09	-0.32	0.21	-0.72, 0.09	-0.25	1.31
	Physical Sciences x Asian	-0.46	0.20	-0.85, -0.08	-0.46	0.22	-0.90, -0.02	0.83	-0.41	0.20	-0.81, -0.02	0.05	1.00
	Physical Sciences x Minority	0.02	0.27	-0.50, 0.55	0.02	0.25	-0.48, 0.52	1.17	-0.22	0.23	-0.66, 0.22	-0.24	1.38
	Social Sciences x Asian	-0.53	0.24	-1.01, -0.05	-0.53	0.25	-1.03, -0.03	0.92	-0.71	0.22	-1.15, -0.28	-0.18	1.19
	Social Sciences x Minority	-0.26	0.24	-0.74, 0.21	-0.26	0.23	-0.71, 0.18	1.09	-0.38	0.20	-0.78, 0.02	-0.12	1.44
	Engineering x Asian	-0.61	0.18	-0.97, -0.25	-0.61	0.21	-1.02, -0.19	0.73	-0.62	0.19	-0.99, -0.25	-0.01	0.90
	Engineering x Minority	-0.24	0.27	-0.77, 0.28	-0.24	0.25	-0.73, 0.24	1.17	-0.39	0.22	-0.82, 0.04	-0.15	1.51
	Non-S & E Fields x Asian	-0.51	0.32	-1.14, 0.11	-0.51	0.32	-1.14, 0.11	1.00	-0.41	0.30	-0.99, 0.17	0.1	1.14
	Non-S & E Fields x Minority	-0.01	0.39	-0.77, 0.75	-0.01	0.39	-0.77, 0.76	1.00	0.03	0.35	-0.66, 0.72	0.04	1.24
	Other Categories x Asian	**-1.70**	**0.83**	**-3.32, -0.08**	**-1.70**	**0.85**	**-3.36, -0.05**	**0.95**	**-1.41**	**0.76**	**-2.90, 0.08**	**0.29**	**1.19**
	Other Categories x Minority	0.93	0.92	-0.87, 2.73	0.93	1.00	-1.03, 2.90	0.85	0.22	0.95	-1.66, 2.09	-0.71	0.94
**2010 SDR /Hours Worked Per Week**	Intercept 1) < = 20	-2.71	0.04	-2.80, -2.62	-2.71	0.05	-2.80, -2.62	0.64	-2.72	0.04	-2.81, -2.63	-0.01	1.00
	Intercept 2) 21–35	-1.87	0.05	-1.96, -1.78	-1.87	0.04	-1.95, -1.79	1.56	-1.89	0.04	-1.96, -1.81	-0.02	1.56
	Intercept 3) 36–40	-0.34	0.04	-0.42, -0.26	-0.34	0.04	-0.41, -0.27	1.00	-0.33	0.04	-0.40, -0.26	0.01	1.00
	**Principal job**	Wald Test: C-S(6) = 420.27, p < 0.0001			Wald Test: C-S(6) = 468.10, p < 0.0001				Wald Test: C-S(6) = 465.51, p < 0.0001				
	Computer and Mathematical Scientists	0.13	0.07	-0.01, 0.27	0.13	0.06	0.01, 0.26	1.36	0.11	0.06	-0.01, 0.23	-0.02	1.36
	Biological, Agricultural and other life scientists	-0.41	0.06	-0.52, -0.30	-0.41	0.05	-0.52, -0.31	1.44	-0.41	0.05	-0.51, -0.31	0.00	1.44
	Physical Scientists	-0.14	0.07	-0.28, 0.01	-0.14	0.06	-0.26, -0.03	1.36	-0.16	0.06	-0.27, -0.05	-0.02	1.36
	Social Scientists	0.50	0.06	0.38, 0.61	0.50	0.05	0.40, 0.59	1.44	0.48	0.05	0.38, 0.57	-0.02	1.44
	Engineers	-0.16	0.06	-0.28, -0.04	-0.16	0.06	-0.28, -0.04	1.00	-0.20	0.06	-0.32, -0.09	-0.04	1.00
	S & E fields	-0.52	0.06	-0.64, -0.40	-0.52	0.06	-0.64, -0.40	1.00	-0.51	0.06	-0.63, -0.39	0.01	1.00
	**Race / Ethnicity**	Wald Test: C-S(2) = 1.89, p = 0.39			Wald Test: C-S(2) = 2.01, p = 0.37				Wald Test: C-S(2) = 2.15, p = 0.34				
	Asian Non-Hispanic only	0.01	0.10	-0.18, 0.19	0.01	0.09	-0.17, 0.17	1.23	-0.01	0.08	-0.17, 0.15	-0.02	1.56
	Under-represented Minorities	0.12	0.09	-0.06, 0.30	0.12	0.09	-0.05, 0.29	1.00	0.11	0.08	-0.04, 0.26	-0.01	1.27
	**Principal job x Race / Ethnicity**	Wald Test: C-S(12) = 46.67, p < 0.0001			Wald Test: C-S(12) = 42.85, p < 0.0001				Wald Test: C-S(12) = 58.19, p < 0.0001				
	Computer and Mathematical Scientists x Asian	0.42	0.12	0.19, 0.65	0.42	0.11	0.20, 0.64	1.19	0.43	0.11	0.22, 0.64	0.01	1.19
	Computer and Mathematical Scientists x Under- represented minorities	-0.19	0.16	-0.49, 0.12	-0.19	0.16	-0.51, 0.13	1.00	-0.21	0.14	-0.49, 0.06	-0.02	1.31
	Biological, Agricultural and other life scientists x Asian	0.31	0.13	0.06, 0.56	0.31	0.11	0.08, 0.53	1.40	0.24	0.11	0.03, 0.45	-0.07	1.40
	Biological, Agricultural and other life scientists x Under-represented minorities	-0.01	0.13	-0.26, 0.23	-0.01	0.13	-0.26, 0.24	1.00	0.02	0.11	-0.20, 0.24	0.03	1.40
	Physical Scientists x Asians	0.34	0.13	0.09, 0.60	0.34	0.13	0.10, 0.59	1.00	0.34	0.12	0.12, 0.57	0.00	1.17
	Physical Scientists x Under-represented Minorities	0.11	0.15	-0.18, 0.40	0.11	0.15	-0.17, 0.40	1.00	0.12	0.13	-0.13, 0.38	0.01	1.33
	Social Scientists x Asians	-0.01	0.16	-0.31, 0.31	-0.01	0.14	-0.27, 0.27	1.31	0.01	0.12	-0.24, 0.25	0.02	1.78
	Social Scientists x Under-Represented Minorities	-0.13	0.12	-0.37, 0.10	-0.13	0.12	-0.36, 0.09	1.00	-0.21	0.1	-0.41, 0.01	-0.08	1.44
	Engineers x Asians	0.39	0.11	0.18, 0.61	0.39	0.11	0.17, 0.61	1.00	0.43	0.11	0.22, 0.63	0.04	1.00
	Engineers x Under-represented Minorities	-0.30	0.16	-0.60, 0.01	-0.30	0.15	-0.59, 0.01	1.14	-0.24	0.13	-0.51, 0.02	0.06	1.51
	S & E fields x Asians	0.42	0.13	0.16, 0.68	0.42	0.13	0.16, 0.68	1.00	0.36	0.12	0.12, 0.60	-0.06	1.17
	S & E fields x Under-represented Minorities	0.22	0.15	-0.07, 0.52	0.22	0.14	-0.06, 0.50	1.15	0.23	0.13	-0.01, 0.48	0.01	1.33
		**Fully Accounting for Complex Design**			**Accounting for Final Weights Only**				**Ignoring all Complex Sampling Features**				
**Survey /Dependent Variable**	**Predictor Variables (Possible Values)**	**Estimated Coefficient**	**R-SE**	**95% CI**	**Estimated Coefficient**	**TSL-SE**	**95% CI**	**Ratio of Variances: R-SE**^**2**^ **/ TSL-SE**^**2**^	**Est. Coef.**	**SRS-SE**	**95% CI**	**Bias of Unweighted Estimate**	**MEFF**
**2010 NSCG /Log-transformed Current Salary**	Intercept	**10.93**	**0.03**	**10.88, 10.98**	**10.93**	**0.03**	**10.88, 10.98**	1.00	**11.10**	**0.01**	**11.07, 11.13**	0.17	9.00
	Female	-0.49	0.04	-0.56, -0.41	-0.49	0.04	-0.56, -0.41	1.00	-0.41	0.02	-0.45, -0.38	0.08	4.00
	S & E Degree	**0.16**	**0.03**	**0.10, 0.22**	**0.16**	**0.03**	**0.10, 0.22**	1.00	**0.02**	**0.02**	**-0.01, 0.05**	-0.14	2.25
	Female x S & E Degree	0.03	0.04	-0.05, 0.12	0.03	0.05	-0.06, 0.12	0.64	0.01	0.02	-0.03, 0.06	-0.02	4.00
**2010 NSCG /Having a Science and Engineering Job**	Intercept	**-0.76**	**0.02**	**-0.80, -0.72**	**-0.76**	**0.03**	**-0.81, -0.70**	0.44	**0.40**	**0.01**	**0.38, 0.43**	1.16	4.00
	Female	-0.28	0.03	-0.35, -0.22	-0.28	0.04	-0.37, -0.20	0.56	-0.40	0.02	-0.43, -0.37	-0.12	2.25
	**Race / Ethnicity**	Wald Test: C-S(6) = 366.37, p < 0.0001			Wald Test: C-S(6) = 231.55, p < 0.0001				Wald Test: C-S(6) = 1803.61, p < 0.0001				
	Asian, Non-Hisp.	**1.19**	**0.07**	**1.05, 1.33**	**1.19**	**0.08**	**1.03, 1.35**	0.77	**-1.22**	**0.05**	**-1.33, -0.43**	-2.41	1.96
	Am. Ind./Al. Nat.	-0.42	0.30	-1.00, 0.16	-0.42	0.38	-1.16, 0.32	0.62	-0.88	0.23	-1.34, -0.43	-0.46	1.70
	Black, Non-Hisp.	**-0.32**	**0.10**	**-0.51, -0.13**	**-0.32**	**0.12**	**-0.55, -0.09**	0.69	**-1.52**	**0.06**	**-1.64, -1.40**	-1.20	2.78
	Hispanic	**-0.23**	**0.09**	**-0.41, -0.05**	**-0.23**	**0.10**	**-0.42, -0.04**	0.81	**-1.58**	**0.06**	**-1.70, -1.46**	-1.35	2.25
	Nat. Haw./Pac. Is.	**0.27**	**0.47**	**-0.65, 1.19**	**0.27**	**0.48**	**-0.66, 1.20**	0.96	**-1.70**	**0.35**	**-2.39, -1.00**	-1.97	1.80
	Multiple Race	**-0.01**	**0.20**	**-0.40, 0.38**	**-0.01**	**0.22**	**-0.43, 0.41**	0.83	**-1.50**	**0.14**	**-1.77, -1.24**	-1.49	2.04
	**Race / Ethnicity x Female**	**Wald Test: C-S(6) = 8.65, p = 0.1944**			**Wald Test: C-S(6) = 8.00, p = 0.2383**				Wald Test: C-S(6) = 2204.15, p < 0.0001				
	Asian, Non-Hisp. x Female	**-0.26**	**0.12**	**-0.49, -0.03**	**-0.26**	**0.13**	**-0.51, -0.01**	0.85	**1.87**	**0.06**	**1.76, 1.99**	2.13	4.00
	Am. Ind./Al. Nat. x Female	0.72	0.53	-0.31, 1.75	0.72	0.57	-0.40, 1.85	0.86	0.91	0.28	0.36, 1.47	0.19	3.58
	Black, Non-Hisp. x Female	**0.13**	**0.14**	**-0.14, 0.40**	**0.13**	**0.16**	**-0.18, 0.44**	0.77	**1.43**	**0.07**	**1.30, 1.57**	1.30	4.00
	Hispanic x Female	**0.01**	**0.13**	**-0.25, 0.27**	**0.01**	**0.14**	**-0.27, 0.29**	0.86	**1.66**	**0.07**	**1.53, 1.80**	1.65	3.45
	Nat. Haw./Pac. Is. x Female	-0.18	0.62	-1.40, 1.04	-0.18	0.65	-1.46, 1.10	0.91	1.83	0.38	1.08, 2.58	2.01	2.66
	Multiple Race x Female	**-0.31**	**0.30**	**-0.90, 0.28**	**-0.31**	**0.29**	**-0.88, 0.26**	1.07	**1.65**	**0.15**	**1.35, 1.94**	1.96	4.00

R-SE, Design-based standard error using replicate weights; TSL-SE, Taylor Series Linearization standard error, recognizing variance in the weights; SRS-SE, Simple Random Sample Standard Error, ignoring final weights and replicate weights; MEFF, Misspecification Effect on variance estimate, ignoring all complex sampling features [34]; C-S, Chi-Square Statistic with degrees of freedom in parentheses; **Boldface cell values**, cases where inferences would change depending on whether one accounted for the complex sampling features; Reference categories in SDR models: White Non-Hispanic (both), S & E Fields (salary), Non S & E Fields (hours per week); Reference category of race/ethnicity for NSCG model of having a science and engineering job: White Non-Hispanic.

First considering the estimated models for the 2010 SDR in Table 7, we find that overall inference related to the importance of the major degree field × race/ethnicity interaction in the model predicting salary greater than $150K would change depending on whether the complex sampling features were taken into account. When fully accounting for the complex sampling features, one would conclude that this interaction is significant (based on the design-adjusted Wald test), and simply accounting for the weights or ignoring the design features entirely would lead to different conclusions all together. Closer inspection of the results reveals that this change in inference is largely due to increased precision of the estimates when accounting for the stratified sample design of the SDR via the replicate weights (which was possible in theory); several ratios of variance estimates based on fully accounting for the complex sampling (versus using the weights only) are less than 1, and this pattern is evident in Fig 4. In this first model, the misspecification effects that would arise when completely ignoring the complex sampling features vary slightly around 1.0 and depend on the estimate (Fig 5).

In the second SDR model, we find that accounting for the complex sampling features does not have a large impact on inferences related to the relationships of principal job category and race / ethnicity with hours worked per week, suggesting that this model was fairly well-specified. Regardless of the analysis approach used, we would conclude that the differences between the race / ethnicity groups in the distribution of hours worked per week clearly depend on the principal job category. We do note that for this model, fully accounting for the complex sampling features tends to result in MEFF values that are greater than 1, suggesting that the SDR stratification resulted in larger gains in the efficiency of the estimated coefficients for the salary model than for the model predicting hours worked per week. In general, failing to account for the complex sampling features in the second model (for hours worked per week) would simply lead to slightly understated standard errors. Avoiding these slight losses in the efficiency of the estimates by ignoring both the respondent weights and the replicate weights may not be problematic if the model was well-specified and the weights are not carrying any information about the estimated coefficients [16, 36].

Next, considering the first NSCG model for log-transformed current salary, we find that a failure to incorporate the final NSCG weights in estimation would lead to completely different inference regarding the main effect of having a science and engineering degree on salary. When ignoring the NSCG weights, there is no evidence of those with a science and engineering degree having a different mean salary from those with a different degree (given the non-significant interaction between gender and type of degree). However, when using the weights in estimation, we see evidence of a much larger positive (and significant) effect of having a science and engineering degree on expected current salary. A failure to account for the weights would thus lead to a completely different conclusion regarding the benefits of having a degree in this area (see Fig 3). We also see evidence of substantially understated standard errors for the estimated regression coefficients when completely ignoring the complex sampling features, with none of the misspecification effects falling below 2.0 (and one as large as 9.0); this pattern is evident in Fig 5. Most of the impact of the complex sampling on the standard errors comes from the variance in the weights, as accounting for the additional complex sampling features via the replicate weights does not lead to substantial changes in the estimated standard errors.

Finally, considering the second NSCG model for the binary indicator of having a job in a science and engineering field, we see that ignoring the weights in estimation leads to substantial changes in the estimates and corresponding inferences. When ignoring the weights in estimation, one would conclude that there is strong evidence of an interaction between gender and race / ethnicity when predicting the probability of having a science and engineering job. When accounting for the weights in estimation, there is no longer evidence of a significant interaction, and the estimated coefficients shift substantially. In addition, we once again see evidence of substantially understated standard errors for the estimated regression coefficients when completely ignoring the complex sampling features, with none of the misspecification effects falling below 1.7 (and one as large as 4.0); see Fig 5. We also see additional evidence of fully accounting for the stratified sampling (via the replicate weights) introducing more efficiency in the estimates relative to just using the weights alone (Fig 4). As was expected, we therefore see consistent evidence in both the SDR and the NSCG of the possible gains in efficiency from fully accounting for the stratified sampling via the replicate weights (relative to using the respondent weights alone), whether generating descriptive estimates or estimating regression models.

## Discussion

We highlight six key findings in this study:

1. The sampled research products rarely accounted for the complex design features of the samples underlying the SESTAT survey data, and these prevalence rates did not vary across the three SESTAT surveys: only 55% of the products incorporated the publicly-available sampling weights into the analyses, only 8% of the products accounted for the complex sampling features when estimating variances, and only 11% of the products presenting design-based analyses performed appropriate subpopulation analyses accounting for the complex sampling [2].
2. Slightly more than half of the sampled products (56%) used design-based (vs. model-based) approaches (especially NSCG products), and while the majority of the products (74%) described results with respect to the target populations of the SESTAT samples (especially SDR products), accounting for sampling weights or using design-based approaches was *not* associated with this method of describing the results.
3. There was no evidence of trends in the prevalence of the different analytic approaches over time.
4. Different types of products did not vary in terms of the prevalence of the approaches used, but peer-reviewed journal articles had the lowest rates of accounting for the complex sampling features when estimating variances.
5. The presence of statistical reviewers on the editorial boards of peer-reviewed journals (44% of the articles published in peer-reviewed journals) increased the probability of accounting for the complex sampling features in analysis.
6. A failure to fully account for the complex sampling features of the SESTAT data sets in analysis has critical implications for inferences related to popular descriptive and analytic (regression) parameters based on these data.

The first two findings are largely consistent with the results of one pilot study of the analytic error problem that examined 100 research products from *different* (i.e., non-SESTAT) surveys [29–30], such as the National Health and Nutrition Examination Survey (NHANES), and provide additional evidence suggesting that secondary analysts may be making analytic errors quite frequently when working with public-use survey data sets. A failure to account for sampling weights in estimation can substantially bias population estimates of key descriptive parameters, and a failure to account for complex sampling features when estimating the variances of estimates can lead to incorrect statements regarding sampling variability. Furthermore, if roughly half of secondary analysts are using model-based approaches to analyze the SESTAT data, these models need to account for the complex sampling features in some way to make sure that they are not informative regarding the estimates of interest, and we rarely found evidence of this approach being used.

The next four findings contribute unique knowledge about the analytic error problem. This study assessed the prevalence of apparent analytic errors in different types of research products (including conference proceedings papers and book chapters), for different subject matter (describing the college-educated science and engineering work force in the U.S.), across multiple decades (with a stratified sample of research products, with decades treated as strata). We also extended prior knowledge related to the problem of analytic error by presenting the implications of actually making analytic errors when using the public-use SESTAT data files (given that selected SESTAT design information is only available upon request), finding several examples of inferences that would change substantially when failing to account for the complex sampling features.

The relatively static prevalence of SESTAT investigators employing “appropriate” analytic approaches over time raises concern about whether there has been sufficient dissemination of knowledge across different fields with regard to appropriate techniques for analyzing survey data. Taken together with 1) the relatively low prevalence of appropriate approaches found in this study and 2) the finding that peer-reviewed articles in journals with dedicated statistical reviewers were more likely to use theoretically appropriate approaches, we feel that reviewers *and* consumers of these research products should take more care in making sure that appropriate methods for survey data analysis have been employed by the study authors. This type of peer feedback can play an important role in dissemination of knowledge about the importance of using these methods to avoid analytic errors when making inferences about larger populations based on survey data. While word limits for academic journals (which we were only able to determine for about half of the peer-reviewed articles) may ultimately lead to the removal of details describing the analytic methods used in a given study, we feel that transparency regarding analytic methods is essential for enabling reproducible research and confirming that a given study has employed analytic techniques appropriate for survey data.

Furthermore, web sites providing guidance for individuals submitting manuscripts should explicitly indicate that any products presenting secondary analyses of complex sample survey data need to demonstrate that they have sufficiently incorporated any available complex sampling features into the analyses presented. As outlined earlier in this paper, there are many theoretically sound design-based and model-based approaches currently available to secondary analysts and possible using standard statistical software (especially design-based methods), so there is no reason that secondary analysts should not be at least considering the effects of these complex sampling features on their analyses in future publications. Adding these restrictions to web sites accepting these types of research products will also help to ensure that analysts are taking all steps to avoid the possibility of making analytic errors. We also encourage faculty and researchers from more applied fields teaching courses on research methods to place more emphasis on analytic techniques for survey data in their courses. This will also help to enhance the dissemination of knowledge regarding appropriate analytic techniques to different fields.

Finally, additional replications of this study using other survey data sources in general would provide more empirical background regarding the magnitude of this problem. The two studies conducted to date (including this one) have analyzed a total sample of 245 scientific products, and while the review and coding of these publications is fairly time-intensive, this is still a very small sample of all research products that have ever presented secondary analyses of complex sample survey data. For example, one could consider other major national surveys focusing on educational subject matter, such as the Programme for International Student Assessment (PISA) or the National Assessment of Educational Progress (NAEP). We used Google Scholar to identify (in a non-random fashion) 10 of the most frequently-cited peer-reviewed journal articles presenting analyses of PISA and NAEP data, just for illustration purposes. We found that among 7 articles presenting analyses of PISA data and 3 articles presenting analyses of NAEP data (see S1 Text), two articles completely ignored the complex sampling features (weights and variance estimation codes) in analyses, one article ignored the weights in estimation but accounted for the complex sampling features in variance estimation, and two articles used incorrect methods for subpopulation analysis. The prevalence of analytic error may well vary across different types of survey programs, and could be a function of the documentation available for secondary analysts or the ease with which one can obtain data describing the complex sampling features. Additional evidence of potential analytic errors in other contexts would further underscore the importance of educating researchers and scientists from other fields about the implications of not performing these analyses correctly, and also considering analytic error as an essential component of the larger Total Survey Error (TSE) framework.

## Supporting Information

S1 Data Coded database for 145 sampled research products. Includes worksheet containing legend for coded variables.
(XLSX)

S2 Data SAS code for performing analyses of SESTAT public-use data files. Includes comments.
(SAS)

S1 Text Complete reference list for all 150 sampled articles, including 10 additional articles presenting analyses of PISA and NAEP data. (DOC)

S2 Text PRISMA check list for evaluation of meta-analyses. (DOC)
