# Supplementary material for: How Big of a Problem is Analytic Error in Secondary Analyses of Survey Data?
Source: PLoS One. 2016 Jun 29;11(6):e0158120. doi: 10.1371/journal.pone.0158120 (PMC4927119; doi:10.1371/journal.pone.0158120)
Supplement: S1 Text — (DOC) [file pone.0158120.s003.doc]

**References (Alphabetical) for 150 Sampled Research Products**

We provide an alphabetical reference list for all 150 sampled research products below, without specific numbering (given that these individual articles are never directly cited in the study, and analyses are only performed in aggregate). We remind readers that the codes assigned to each article (see S1 Data) are based entirely on the information presented in the article; word count limits certainly may have prevented the authors from providing more details regarding the analysis approach. This is why we focus on “apparent” analytic errors when describing the study results.

Also included in this list are the 10 additional articles mentioned in the Discussion section that presented analyses of data from the PISA and NAEP surveys.

Agarwal, R., & Ohyama, A. (April 1, 2013). Industry or academia, basic or applied? Career choices and earnings trajectories of scientists. *Management Science, 59*, 4, 950-970.

Åkerblom, M. (1999). Mobility of highly qualified manpower: A feasibility study on the possibilities to construct internationally comparable indicators; report for Camire (Eurostat) and the OECD, 14 December 1999. Paris: OECD.

Altonji, J. G., Kahn, L. B., & Speer, J. D. (January 1, 2014). Trends in Earnings Differentials across College Majors and the Changing Task Composition of Jobs. *American Economic Review, 104*, 5, 387-393.

Ammermueller, A. (2007). Poor background or low returns? Why immigrant students in Germany perform so poorly in the programme for international student assessment. *Education Economics*, 15(2), 215-230.

Astebro, T., Bazzazian, N., & Braguinsky, S. (May 1, 2012). Startups by recent university graduates and their faculty: Implications for university entrepreneurship policy. *Research Policy, 41*, 4, 663-677.

Auriol, L ., Sexton, J., & Bordt, M.(2002). International Mobility of the Highly Skilled. Collection of papers prepared for the Seminar on International Mobility of Highly Skilled Workers in Paris, France, June 11-12, 2001.

Baker, J. G. (January 1, 2002). The influx of women into legal professions: an economic analysis. *Monthly Labor Review*, *125*, 14-24.

Baker, J. G., & Jorgensen, B. K. (January 1, 2000). Leaving the Law: Occupational and Career Mobility of Law School Graduates. *Journal of Legal Education, 50*, 16-34.

Bedard, K. and Herman, D. (April 1, 2008). Who goes to graduate/professional school? The importance of economic fluctuations, undergraduate field, and ability. *Economics of Education Review, 27*, 2, 197-210.

Bender, K. A., & Heywood, J. S. (May 1, 2006). Job Satisfaction of the Highly Educated: The Role of Gender, Academic Tenure, and Earnings. *Scottish Journal of Political Economy, 53*, 2, 253-279.

Bernard, J. (March 1, 2008). The migration of researchers from central Europe during the transition period. *Sociologia, 40*, 3, 191-214.

Black, D., Haviland, A., Sanders, S., & Taylor, L. (January 1, 2006). Why Do Minority Men Earn Less? A Study of Wage Differentials among the Highly Educated. *The Review of Economics and Statistics, 88*, 2, 300-313.

Black, D. A., Haviland, A., Sanders, S., & Taylor, L. J. (January 1, 2008). Gender Wage Disparities among the Highly Educated*. Journal of Human Resources, 43*, 3, 630-659.

Blume-Kohout, M.E., & Clack, J.W. (Dec 23, 2013). Are graduate students rational? Evidence from the Market for Biomedical Scientists. *PLoS ONE* 8(12): e82759. doi:10.1371/journal.pone.0082759.

Bound, J., Demirci, M., Khanna, G., Turner, S. E., & National Bureau of Economic Research, (2014). Finishing degrees and finding jobs: U.S. higher education and the flow of foreign IT workers.

Brown,C., & Freeman,E. (August 30, 2013). Estimating net costs of Adopting Family Friendly Policies at Universities. Description of a similar program. Working paper series retrieved from Institute for Research on Labor and Employment website: <http://escholarship.org/uc/item/6gw3621r>.

Brown, S.V. (1988). Increasing Minority Faculty: An Elusive Goal. Educational Resources Information Center (ERIC), Number ED299904.

Bushery, J. M., Brick, J.M ., Severynse, J., & McGuinness, R.A. (1996). How interview mode affects data reliability. Washington, DC: US Census Bureau.

Carroll, T., Assane, D., & Busker, J. (January 1, 2014). Why It Pays to Major in Economics*. Journal of Economic Education, 45*, 3, 251-261.

Cech, E. A. (June 1, 2013). Ideological Wage Inequalities? The Technical/Social Dualism and the Gender Wage Gap in Engineering. *Social Forces, 91*, 4, 1147-1182.

Clewell, B. C., & National Science Foundation (U.S.). (2006). Revitalizing the nation's talent pool in STEM: Science, technology, engineering, and mathematics. Washington, DC: The Urban Institute.

Clewell, B.C., De cohen, C.C., Tsui,L., & Deterding, N.( 2006). Revitalizing the talent pool in STEM. Washington, DC: The Urban Institute.

Clewell, B.C., De cohen, C.C., Tsui,L., Forcier, L., Gao,E., Young, N., Deterding, N., & West, C. (November 2005). Evaluation of the National Science Foundation Louis Stokes Alliances for Minority Participation Program. Washington, DC: The Urban Institute.

Collins, S. M. (May 1, 2000). Minority Groups in the Economics Profession. *Journal of Economic Perspectives, 14*, 2, 133-148.

Corley, E. A., & Sabharwal, M. (December 1, 2007). Foreign-born academic scientists and engineers: producing more and getting less than their U.S.-born peers?. Research in Higher Education: *Journal of the Association for Institutional Research, 48*, 8, 909-940.

Craft, R. K., & Baker, J. G. (September 6, 2003). Do Economists Make Better Lawyers? Undergraduate Degree Field and Lawyer Earnings. *The Journal of Economic Education, 34*, 3, 263-281.

Cromley, J. G. (2009). Reading Achievement and Science Proficiency: International Comparisons from the Programme on International Student Assessment. *Reading Psychology*, 30(2), 89-118.

Decohen, C.C. (2006). Building the nation's scientific capacity: Evidence from the Louis Strokes Alliances for Minority Participation program. Washington, DC: The Urban Institute.

Del, R. A. F., & Hersch, J. (January 1, 2008). Double your major, double your return? *Economics of Education Review, 27,* 4, 375-386.

Delen, E., & Bulut, O. (2011). The relationship between students' exposure to technology and their achievement in science and math. *TOJET: The Turkish Online Journal of Educational Technology*, 10(3).

Edwards, R. D. (December, 2004). Are the Wise also wizened? Population aging and knowledge production. Prepared for the December 2004 Symposium on Population Ageing and Economic Growth hosted by Vienna Institute of Demography and the international Institute for Applied System analysis, Vienna Austria.

Edwards, R. D. (January 1, 2008). Declining Mortality among British Scientists during the Age of Enlightenment. *Population and Development Review, 34*, 103-125.

Elfenbein, D. W., Hamilton, B. H., & Zenger, T. R. (March 2008). The entrepreneurial spawning of scientists and engineers: Stars, Slugs and the small firm effect. Paper presented at the research Symposium on the Economics and Law of the entrepreneur at the Searle Center Northwestern Law Chicago, IL.

Elfenbein, D. W., Hamilton, B. H., & Zenger, T. R. (April 1, 2010). The small firm effect and the entrepreneurial spawning of scientists and engineers. *Management Science, 56*, 4, 659-681.

Fesco, R. S., Broach, R. J., & Grigorian, K. H. (2003). Data that do not persuade: Results of an Incentive Experiment. Washington, D.C: National Science Foundation. Chicago, IL: National Opinion Research Center.

Fogg, N. P., Harrington, P.E. (March 2012). The earnings of foreign educated college graduates An examination of the determinants of the hourly Earnings of College educated Immigrants. (Prepared Under Contract No. ED-VAE-11-O-0018).Philadelphia, PA: Center for Labor Markets and Policy.

Fogg, N. P., Harrington, P.E. (March 2012). Involuntary Part-Time Employment Problems among College-Educated Immigrants in the United States.( Prepared Under Contract No. ED-VAE-11-O-0018). Philadelphia, PA: Center for Labor Markets and Policy.

Foley, D. J. (September 2009). Characteristics of Doctoral Scientists and Engineers in the United States: 2006. Detailed Statistical Tables NSF 09-317. Arlington, VA: National Science Foundation, Division of science resources statistics.

Frehill, L. M. (2007). What do women do with engineering degrees? WEPAN-Women in Engineering Program and Advocates Network.

Frehill, L. M. (2012). Gender and Career Outcomes of U.S Engineers. *International Journal of Gender, Science and Technology, 4*, 149-166.

Gemici, A. and Wiswall, M. (February 1, 2014). Evolution Of Gender Differences In Post-Secondary Human Capital Investments: College Majors. *International Economic Review, 55*, 1, 23-56.

Ginther, D. K., & Kahn, S. (September 1, 2004). Women in Economics: Moving Up or Falling Off the Academic Career Ladder? *Journal of Economic Perspectives, 18*, 3, 193-214.

Ginther, D. K., Liu, F., Masimore, B., Schaffer, W. T., & Schnell, J. (2009). Diversity in academic biomedicine: An evaluation of education and career outcomes with implications for policy. Lawrence, Kan: Univ. of Kansas, Dep. of Economics.

Ginther, D. K., & Rasier, D. G. (2003). Work activities as Firm-Specific Human Capital: Estimates of the Effects on Wages. Lawrence, KS: University of Kansas.

Glover, J. (2001). Report on study visit to Washington DC by Judith Glover for the Women and Science unit, Science and Society directorate, Research Directorate General, European Commission.

Gottlieb, P. D., & Joseph, G. (October 1, 2006). College-To-Work Migration of Technology Graduates and Holders of Doctorates within the United States. *Journal of Regional Science, 46*, 4, 627-659.

Goulden, M., Mason, M., & Frasch, K. (November 1, 2011). Keeping Women in the Science Pipeline. *The Annals of the American Academy of Political and Social Science, 638*, 1, 141-162.

Graham, J. and Smith, S. (June 1, 2005). Gender differences in employment and earnings in science and engineering in the US. *Economics of Education Review, 24*, 3, 341-354.

Greenman, E. (January 1, 2011). Asian American-White Differences in the Effect of Motherhood on Career Outcomes. *Work and Occupations, 38*, 1, 37-67.

Haelen, A., Jang, D., Lan, F., & Baylor, Amy. (2013). Assessing Survey Quality through streamlined data processing. Washington, D.C: Mathematica Policy Research, National Science Foundation. Minnetonka, Minnesota: United Healthcare.

Haertel, G. D., Walberg, H. J., Junker, L., & Pascarella, E. T. (1981). Early adolescent sex differences in science learning: Evidence from the National Assessment of Educational Progress. *American Educational Research Journal*, 18(3), 329-341.

Halonen, J.S. (February 5th, 2011). Executive summary of white paper: Are there too many psychology majors? Pensacola, Fl: University of Florida.

Hanson, S. L., & Fang, F. (January 1, 2009). Race, Sex, and Job Satisfaction in Science Occupations: A Focus on Asian-Americans*. Journal of Women and Minorities in Science and Engineering, 15*, 4, 357-377.

Hao, L. (September 1, 2013). Admission-Group Salary Differentials in the United States: The Significance of the Labour-Market Institutional Selection of High-Skilled Workers. *Journal of Ethnic and Migration Studies, 39*, 8, 1337-1360.

Hemelt, S. W. (June 1, 2010). The College Double Major and Subsequent Earnings. *Education Economics, 18,* 2, 167-189.

Hersch, J. (December 1, 2013). Opting out among women with elite education. *Review of Economics of the Household, 11*, 4, 469-506.

Hersch, J. (July 2014). Catching up is hard to do: Undergraduate Prestige, Elite Graduate Programs, and the Earnings Premium. Vanderbilt University Law School, working paper number 14-23.

Huang, C. (October 2010). The impact of high-skilled immigration on wages of U.S natives. Paper presented at Population Association of America at an annual meeting in Dallas, Texas, April 2010.

Hunt, J. (February 2010). Which Immigrants are Most Innovative and Entrepreneurial? Distinctions by Entry Visa. IZA Discussion Paper Series, #4745.

Hunt, J., Garant, J.-P., Herman, H., & Munroe, D. J. (March, 2012). Why Don’t Women Patent? Working Paper Series, 17888.

Hunt, J. (September 2012). Why do women leave science and engineering? Discussion Paper Series, Forschungsinstitut zur Zukunft der Arbeit, No. 6885.

Jang, D., & Lin, X. (2007). Evaluation of the National Survey of Recent College Graduates (NSRCG) Postsecondary Institution Sample. Papers Presented at the ICES-III, June 18-21, 2007, Montreal, Quebec.

Jang,D., Lin, X., & Kang, K. (2008). Non-response bias analysis using reluctant respondent in the 2003 National Survey of Recent College Graduates. Washington, D.C: Mathematica Policy Research. Arlington, VA: National Science Foundation.

Jang, D., Sukasih, A., Lin, X., Hang, K., & Cohen, S. (2009). Effects of misclassification of Race/Ethnicity categories in sampling stratification on Survey Estimates. Washington, D.C: Mathematica Policy Research. Arlington, VA: National Science Foundation.

Jang, D., Zhao, Z., & Hang, K. (2009). Analyzing the effects of data collection mode change in the 2003 National Survey of Recent College Graduate. 2009 American Association for Public Opinion Research Annual Conference in Hollywood, FL, May 15^th^, 2009.

Kahn, S. & Ginther, D. K. (September 10, 2012). Postdoctoral Training and the Career Outcomes of Biomedical PhD's. Lawrence, Kan: Univ. of Kansas, Department of Economics.

Kahn, S., La Matina, G., MacGarvie, M., & Ginther, D. (2013). "Hobos", "Stars" and Immigrant Entrepreneurship. Boston, MA: Boston University. Lawrence, KS: University of Kansas.

Kennedy, T. J. (October 1994).Graduate education in the Biomedical Sciences: Critical Observations on Training for Research Careers. *Academic Medicine, 69*, 10, 779-799.

Kim, C. H., & Sakamoto, A. (January 1, 2010). Have Asian American Men Achieved Labor Market Parity with White Men?. *American Sociological Review, 75*, 6, 934-957.

Kim, D., Twombly, S., & Wolf-Wendel, L. (December 7, 2012). International Faculty in American Universities: Experiences of Academic Life, Productivity, and Career Mobility. *New Directions for Institutional Research, 155*, 155, 27-46.

La Matina, G., Ginther, D., & Kahn, S., MacGarvie , M. (no date). Immigrants and Science Entrepreneurship. Boston, Ma: Boston University. Lawrence, KS: University of Kansas.

Lan, X. (December 7, 2013). The Effects of Green Cards on the Wages and Innovations of New PhDs. *Journal of Policy Analysis and Management, 32*, 4, 807-834.

Lee, Y. & Sabharwal, M. (no date). Attributes of Job satisfaction across the public, nonprofit and for profit sectors: Survey of recent college graduates in science, engineering and health fields. Dallas, TX: University of Texas at Dallas.

Lee, Young-Joo & Sabharwal, M. (September 16, 2014). Education–Job Match, Salary, and Job Satisfaction across the Public, Non-Profit, and For-Profit Sectors: Survey of recent college graduates. *Public Management Review*, DOI:10.1080/14719037.2014.957342.

Levin, S. G., Black, G.C., Winkler, A.E., & Stephan, P.E. (2002). The changing career outcomes of Citizen and Non-Citizen Scientist and Engineers in Higher Education. Paper presented at the Higher Education Research Group Meeting at the National Bureau of Economic Research.

Levin, S. G., & Stephan, P. E. (October 1, 1989). Age and research productivity of academic scientists. *Research in Higher Education, 30*, 5, 531-549.

Levin, S. G., Black, G. C., Winkler, A. E., & Stephan, P. E. (September 1, 2004). Differential Employment Patterns for Citizens and Non-Citizens in Science and Engineering in the United States: Minting and Competitive Effects. *Growth and Change, 35*, 4, 456-475.

Levin, S. G., & Stephan, P.E. (January 1, 2005). Women and Underrepresented Minorities in the IT Workforce. *Journal of Women and Minorities in Science and Engineering, 11*, 4, 345-364.

Lowell, B. L., & Avato, J. (January 1, 2014). The wages of skilled temporary migrants: Effects of visa pathways and job portability. *International Migration, 52*, 3, 85-98.

Madsen, P. E. (January 1, 2013). The Integration of Women and Minorities into the Auditing Profession since the Civil Rights Period. *Accounting Review, 88*, 6, 2145-2178.

Malcom, L. E. (Winter 2010). Charting the pathways to STEM for latina/o students: The role of community colleges. *New Directions for Institutional Research, 2010, 148*, 29-40.

Malcom, L. E., & Dowd, A. (2003). College student debt as opportunity or disadvantage? A reconceptualization and Application to STEM Graduate enrollment. Riverside, CA: University of California Riverside. Los Angeles, CA: University of Southern California.

Malcom, L. E., & Dowd, A. C. (Winter 2012). The Impact of Undergraduate Debt on the Graduate School Enrollment of STEM Baccalaureates. *Review of Higher Education, 35*, 2, 265-305.

Malcom, L. E., Dowd, A. C. & Yu, T. (November 2010). Tapping HIS-STEM funds to improve Latina and Latino Access to STEM professions. Los Angeles, CA: University of Southern California Center for Urban Education.

Malcom-Piqueux, L. (January 1, 2015). Application of Person-Centered Approaches to Critical Quantitative Research: Exploring Inequities in College Financing Strategies. *New Directions for Institutional Research, 2014, 163*, 59-73.

Marks, G. (2005). Accounting for immigrant non-immigrant differences in reading and mathematics in twenty countries. *Ethnic and Racial Studies*, 28(5), 925-946.

Marks, G. N. (2008). Accounting for the Gender Gaps in Student Performance in Reading and Mathematics: Evidence from 31 Countries. *Oxford Review Of Education*, 34(1), 89-109.

Martin, A. J., Liem, G. A., Mok, M., & Xu, J. (2012). Problem solving and immigrant student mathematics and science achievement: Multination findings from the Programme for International Student Assessment (PISA). *Journal of Educational Psychology*, 104(4), 1054.

Mason, M. A., & Goulden, M. (November 1, 2004). Marriage and Baby Blues: Redefining Gender Equity in the Academy. *The Annals of the American Academy of Political and Social Science, 596*, 1, 86-103.

Maxfield, B. & Belisle, M. (1985). Science, Engineering, and Humanities Documents in the U.S.: 1983 profile. National Academy Press: Washington, DC.

McDonald, J. T., Warman, C., & Worswick, C. (2011). Immigrant selection systems and occupational outcomes of international medical graduates in Canada and the United States. Kingston, Ontario: Queen's Economics Dep., Queen's University.

McTeague, J. P. (1988). Engineering Personnel Data needs for 1990's. National Academy Press: Washington, DC.

Metcalf, H. (2011). Formation and representation: Critical analyses of identity, supply and demand in science and technology, engineering and mathematics. Tucson, AZ: University of Arizona.

Metcalf, H. E. (January 1, 2014). Disrupting the Pipeline: Critical Analyses of Student Pathways through Postsecondary STEM Education. *New Directions for Institutional Research, 2013, 158*, 77-93.

Michelmore, K . & Sassler, S. (2015). Explaining the gender gap in Earning in STEM: Does group size matter? Ann Harbor: University of Michigan. Ithaca, NY: Cornell University.

Milam, J. (May 1998). The Glut of PhD’s - Complex models for the faculty workforce. Paper presented at the 38th Annual Forum of the Association for Institutional Research held at Minneapolis, Minnesota, May 17-20 ,1998.

Millar, M. M. (June 1, 2013). Interdisciplinary research and the early career: The effect of interdisciplinary dissertation research on career placement and publication productivity of doctoral graduates in the sciences. *Research Policy, 42*, 5, 1152-1164.

Mishagina, N. (January 2008). Career Dynamics of Doctoral Scientists and Engineers. Montreal, Quebec: Analysis Group Inc.

Mishagina, N. (August 10, 2009). Empirical Analysis of Career Transitions of Sciences and Engineering Doctorates in the US. Working paper from Queen's University: Ontario, Canada.

Moonesinghe, R., Mitchell, S., & Pasquini, D. (1995). An identification study of nonrespondents to the 1993 Survey of Doctorate recipients. Washington, DC National Research Council.

Mooney, G. M., Carlson, B.L. (August 30, 1996). Reducing mode effects in "Mark all that apply questions”. Princeton, New Jersey: Mathematica Policy Research.

Mooney, G. M. & Foley, D.J. (July 2011). Community Colleges: Playing an important role in the education of Science, Engineering and Health Graduates. Washington, D.C: National Science Foundation.

Morgan, L.A. (October 1, 2008). Major Matters: A Comparison of the Within-Major Gender Pay Gap across College Majors for Early-Career Graduates. *Industrial Relations, 47*, 4, 625-650.

Morgan, R. P., Kruytbosch, C., & Kannankutty, N. (January 01, 2001). Patenting and Invention Activity of U.S. Scientists and Engineers in the Academic Sector: Comparisons with Industry. *The Journal of Technology Transfer, 26*, 173-183.

Ohyama, A. (2013). Entrepreneurship and job relatedness of Human Capital. Sapporo, Japan: Hokkaido University.

Parker, L. (March, 2004). The Education and Employment of Engineering Graduates. Washington, DC: National Science Foundation.

Parsons, N. L. & Dillman, D.A. (February 2008). Alternative questions for reporting the time periods during which NSRCG respondents took community college courses: A cognitive evaluation. Pullman, Wa: Social and Economic Sciences Research Center.

Pierzchala, M., Wright, D., Wilson, C. & Guerino, P. (2005). Telephone Collection as part of a Multimode Survey. Washington,DC: Mathematica Policy Research. Arlington,VA: Insight Policy Research. Washington, DC: Education Statistics Services Institute.

Prasad, S. (June 1, 2009). Task assignments and incentives: generalists versus specialists. *Rand Journal of Economics, 40*, 2, 380-403.

Preston, A. (Spring 2006). Women leaving science. Haverford, PA: Haverford College.

Prokos, A. H., Padavic, I., & Schmidt, S. A. (October 1, 2009). Nonstandard work arrangements among women and men scientists and engineers. *Sex Roles, 61*, 9, 653-666.

Proudfoot, S. (2011). Characteristics of scientists and engineers in the United States: 2006 [Electronic version]. Arlington, VA: National Center for Science and Engineering Statistics.

Rajecki, D., & Borden, V. (January 01, 2011). Psychology Degrees: Employment, Wage, and Career Trajectory Consequences. *Perspectives on Psychological Science, 6*, 4, 321-335.

Regrets, M.C. (September 2011). Research and Policy Issues in High Skilled International Migration: A perspective with data from the United States. IZA. Discussion Papers no 366.

Ritter, J. A. & West,K. L. (October 2014). Field of study and Earnings Inequality among the highly educated: 1993-2010. Minneapolis, Minnesota: Minnesota Population Research Center.

Robst, J. (August 1, 2007). Education and job match: The relatedness of college major and work. *Economics of Education Review, 26*, 4, 397-407.

Robst, J. & Vangilder, J. (2010). Functional Impairment and the Choice of College Major*. Eastern Economic Journal, 36*, 174-176.

Sabharwal, M. (2008). Categorization of minority groups in Science and Engineering. *Journal of Women and Minorities in Science and Engineering, 14*, 427-446.

Sabharwal, M. (April 17, 2013). Comparing Research Productivity across Disciplines and Career Stages. *Journal of Comparative Policy Analysis:* *Research and Practice, 15*, 2, 141-163.

Sabharwal, M. & Corley, E. A. (September 1, 2009). Faculty job satisfaction across gender and discipline. *Social Science Journal, 46,* 3, 539-556.

Sabharwal, M., Varma, R. (June 2012). Why one leaves? Return Migration of Academic Scientists and Engineers from the United States to India. Paper submitted to 9th IZA annual migration meeting in Bonn, Germany.

Sauermann, H. (June 2013). Fire in the belly? Employee Motives and Innovative Performance in startups versus established Firms. Scheller College of Business, Georgia Tech: Atlanta, Georgia.

Sell, B.C. (2013). Educational Mismatch and Entry into Entrepreneurship. Paper to be presented at 35th DRUID Celebration Conference 2013, Barcelona, Spain Jun 17-19.

Seltzer, N. & Blair, L.M. (1996). Labor Market Trends for Nuclear Engineers through 2005. Oakridge Institute for Science and Education, Oakridge, Tennessee.

Shapira, P. & Kuhlman, S. (January 1, 2003). Learning from science and technology policy evaluation. Northampton, MA: Edward Elgar Publishing.

Sharpe, R. V., & Swinton, O. H. (July 30, 2012). Beyond Anecdotes: A Quantitative Examination of Black Women in Academe. *Review of Black Political Economy, 39*, 3, 341-352.

Shauman, K. A. (September 1, 2009). Are there sex differences in the utilization of educational capital among college-educated workers?. *Social Science Research, 38*, 3, 535-571.

Shettle, C., & Gaddy, C. (November 1, 1998). The Labor Market for Statisticians and other Scientists. *The American Statistician, 52*, 4, 295-302.

Stack, S. (December 1, 2004). Gender, Children and Research Productivity. *Research in Higher Education, 45,* 8, 891-920.

Stephan, P. E. (January 1, 2010). The "I"s Have It: Immigration and Innovation, the Perspective from Academe. *Innovation Policy and the Economy, 10*, 1, 83-127.

Stephan, P., & El-Ganainy, A. (January 1, 2007). The entrepreneurial puzzle: explaining the gender gap. *The Journal of Technology Transfer, 32*, 5, 475-487.

Stephan, P., Gurmu, S., Sumell, A., & Black, G. (2007). Who's patenting in the University? Evidence from the Survey of Doctorate Recipients. *Economics of Innovation and New Technology, 16*, 2, 71-99.

Stephan, P. E., & Levin, S. G. (January 1, 2001). Exceptional contributions to US science by the foreign-born and foreign-educated. *Population Research and Policy Review, 20*, 59-79.

Stephan, P. E., & Levin, S. G. (October 1, 2005). Leaving Careers in IT: Gender Differences in Retention. *The Journal of Technology Transfer, 30*, 4, 383-396.

Stephan, P., Scelatto, G., & Franzoni, C. (May 14, 2014). International Competition for PhDs and Postdoctoral Scholars: What does and does not matter. Paper presented for the NBER Innovation policy and the economy conference in Washington, DC, April 8^th^, 2014.

Suggate, S. P. (2009). School entry age and reading achievement in the 2006 Programme for International Student Assessment (PISA). *International Journal of Educational Research*, 48(3), 151-161.

Sukasih, A., Jang, D., Vartivarian, S., Cohen, S., & Zhang, F. (2009). A simulation Study to compare weighting methods for Nonresponses in the National Survey of Recent College Graduates. Mathematica Policy Research: Washington, DC. National Science Foundation: Arlington, VA.

Sweeper, D., & Smith, S. A. (January 1, 2010). Assessing the Impact of Gender and Race on Earnings in the Library Science Labor Market. *College and Research Libraries, 71,* 2, 171-183.

Takei, I. (January 1, 2012). Are Asian Americans Disadvantaged by Residing More in the West? Migration, Region, and Earnings among Asian American Men. *Sociology Mind, 2*, 2, 158-168.

Tao, Y. (2009). Earnings of Asian Immigrant Computer Scientist: The effect of degree origin. Stevens Institute of Technology: Hoboken, NJ.

Tao, Y. (2010). Multiples Disadvantages? The earnings of Asian Women computer scientists in the United States. *International Journal of Gender, Science and Technology, 2*, 2, 137-158.

Tao, Y. (2011). The Earnings of Asian Engineers in the United States: Race, Nativity, Degree Origin, and Influences of Institutional Factors on Human Capital and Earnings. *Journal of Women and Minorities in Science and Engineering, 17*, 3, 225-249.

Tarrant, S.A. (2001). Predicting Retention of Recent College graduates in science and engineering: Implications for states and organizational recruiting practices. North Carolina State University: Raleigh, NC.

Taylor, D. (January 1, 2007). Employment Preferences and Salary Expectations of Students in Science and Engineering. *Bioscience, 57*, 2, 175-185.

Tong, Y. (February 23, 2010). Place of education, gender disparity, and assimilation of immigrant scientists and engineer’s earnings. *Social Science Research, 39*, 4, 610-626.

Tornatzky, L.G., Gray, D., Tarant, S.A., & Howe, J. (1998). Maine's Science and Engineering Brain Drain: How Much and Why? *Maine Policy Review, 7*, 1 , 1-8.

Turk-Bicakci, L., Berger, A., & Haxton, C. (April1, 2014). The non-academic careers of the stem PHD holders. American Institute for Research. Washington, D.C.

Von Secker, C. (2004). Science achievement in social contexts: Analysis from national assessment of educational progress. *The Journal of Educational Research*, 98(2), 67-78.

Walstad, W. B., & Buckles, S. (2008). The national assessment of educational progress in economics: Findings for general economics. *The American Economic Review*, 98(2), 541-546.

Webber, K. L. (2013). Cultivating the future of Graduate Education Factors Contributing to Salary for Recent Doctorate Degree Recipients. *Planning for Higher Education Journal, 4*, 1-4.

Weinberger, C., & Kuhn, P. (2004).The Evolution of the US Gender Earnings Gap, 1969-1999: A Cohort-Based Analysis. Using NSF and other data creatively to analyze the SE job market May 28 2004.

Whittington, K. B. (2009). Patterns of Male and Female Scientific Dissemination in Public and Private Science. Chapter in NBER book Science and Engineering Careers in the United States: An Analysis of Markets and Employment (p.195-228) National Bureau of Economic Research.

Wolfinger, N.H., Mason, M.A., & Goulden, M. (July 27, 2006). Dispelling the pipeline myth: Gender, Family Formation, and Alternative Trajectories in the Academic Life Course. Working paper from Institute of Public and International Affairs University of Utah.

Xie, B. (2014). Are High Skilled Immigrants really paid low in the US? Department of Economics. Rutgers University.

Yang, L., & Webber, K. L. (January 30, 2015). A decade beyond the doctorate: the influence of a US postdoctoral appointment on faculty career, productivity, and salary. *Higher Education*, DOI: 10.1007/s10734-015-9860-3.

Zanutto, E. (2002). A comparison of propensity score and linear regression analyses of gender gaps in computer systems analyst careers. Philadelphia, PA: University of Pennsylvania.

Zeng, Z. (January 1, 2011). The myth of the glass ceiling: Evidence from a stock-flow analysis of authority attainment. *Social Science Research*, 40(1), 312-325.

Zeng, Z., & Xie, Y. (January 1, 2004). Asian-Americans' Earnings Disadvantage Reexamined: The Role of Place of Education. *The American Journal of Sociology*, 109(5), 1075.

Zhang, F. (November 2010). Incentive experiments: NSF Experiences. Arlington, VA: National Science Foundation.

Zheng, Z. (March 2, 2008). Is there a glass ceiling effect? Evidence from a Stock Flow Analysis. University of Wisconsin-Madison.
